# Synthesis of polycyclic aromatic quinones by continuous flow electrochemical oxidation: anodic methoxylation of polycyclic aromatic phenols (PAPs)

**DOI:** 10.3762/bjoc.20.153

**Published:** 2024-07-24

**Authors:** Hiwot M Tiruye, Solon Economopoulos, Kåre B Jørgensen

**Affiliations:** 1 Department of Chemistry, Bioscience and Environmental Engineering, Faculty of Science and Technology, University of Stavanger, P.O Box 8600 Forus, N-4036 Stavanger, Norway; 2 Advanced Optoelectronic Nanomaterials Research Unit, Department of Chemistry, Norwegian University of Science and Technology, 7491 Trondheim, Norway

**Keywords:** acetal formation, cyclic voltammetry, flow electrochemistry, green oxidation, polycyclic aromatic hydrocarbons

## Abstract

The electrochemical oxidation of polycyclic aromatic phenols (PAPs) has been developed in a microfluidic cell to synthesize polycyclic aromatic quinones (PAQs). Methanol was used as nucleophile to trap the phenoxonium cation formed in the oxidation as an acetal, that later were hydrolysed to the quinone. Formation of hydrogen gas as the cathode reaction caused challenges in the flow cell and were overcome by recycling the reaction mixture through the cell at increased flow rate several times. The specific quinones formed were guided by the position of an initial hydroxy group on the polycyclic aromatic hydrocarbon. An available *para*-position in the PAPs gave *p*-quinones, while hydroxy groups in the 2- or 3-position led to *o*-quinones. The substrates were analysed by cyclic voltammetry for estitmation of the HOMO/LUMO energies to shed more light on this transformation. The easy separation of the supporting electrolyte from the product will allow recycling and makes this a green transformation.

## Introduction

Quinones and their derivatives are applied in various fields such as chemical, environmental, and pharmaceutical industries [1–4]. Their cyclic diketone structures can easily transform into intramolecular unsaturated structures, and their distinct physical properties make them privileged structures in medicinal chemistry [2]. Benzoquinone and naphthoquinone can exist as *ortho*-quinone and *para*-quinone, with the latter considered more stable [5]. Additionally, *p*- and *o*-quinones are formed in metabolism of drugs [6] as well as polycyclic aromatic hydrocarbons (PAHs) by cytochrome P450 (CYP) and other metabolic enzymes [7–8]. Main metabolic pathways form quinone isomers of benzo[*a*]pyrene [8], naphthalene [9–10], and benzene [11].

Numerous methods for the oxidation of phenols or their derivatives to quinones have been described [12]. Oxidation with Fremy’s radical (potassium nitrosodisulfonate) [13] or catalytic systems like methyltrioxorhenium(VII) (MeReO_3_) [14] and 2-iodobenzenesulfonic acids (IBS)/Oxone^®^ [15] led to either *p*-quinones or *o*-quinones, depending on the substituents in the *para*-position to the hydroxy group. Recently, hypervalent iodine reagents have been explored for the oxidation of polycyclic aromatic phenols (PAPs). Oxidation of 1-naphthol derivatives by bis(trifluoroacetoxy)iodobenzene (BTI) furnished *p*-naphthoquinones [16]. Other PAPs follow the same pattern forming *p*-quinones or *o*-quinones when the *para*-position is structurally blocked like in 2-naphthol (**1a**) [17]. Oxidation with iodoxybenzoic acid (IBX) [17] or stabilised IBX (SIBX) [18] form *o*-quinones selectively, even when the *p*-quinones are structurally feasible. However, all these methods constitute toxic hazards and/or produce stoichiometric amounts of waste products making them less desirable for industrial scale [19].

Electrochemical synthesis methods have a huge potential and this field is currently undergoing a renaissance [20–24]. Replacing chemical oxidants with electric current reduces waste production and gives a sustainable and inherently safe alternative to classical synthesis [25–28]. Electrochemical oxidation reactions are further used to emulate enzymatic oxidations of drugs and explore potential metabolites [29–31]. Electrochemical flow systems provide fast electrosynthesis with low cell resistance, large electrode area, and good control of the current [32–34].

**Scheme 1:**
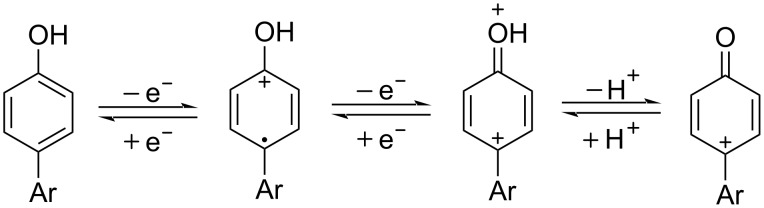
Formation of phenoxonium cation in the anodic oxidation of phenol performed under neutral or weakly basic conditions.

**Scheme 2:**
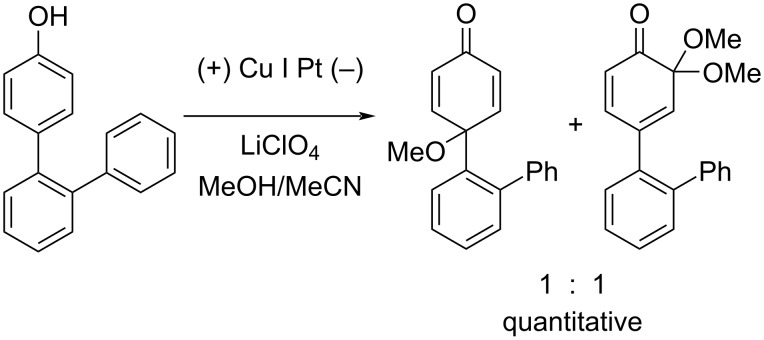
Anodic oxidation reported by Swenton et al. [37].

Early studies on the electrochemical oxidation of phenols revealed that the oxidation passes through a phenoxonium ion and forms acetals in methanol but quinones in the presence of water [35–37]. However, the reaction is sometimes accompanied by the formation of dimers, which indicates a radical intermediate [36]. Swenton and co-workers [37] established evidence for the phenoxonium ion (Scheme 1), and were further able to divert the reaction into forming *ortho*-oxidation due to steric hindrance (Scheme 2). Cyclic voltammetry studies of the oxidation of 2-naphthol (**1a**) into *o*-quinone **5** revealed that the oxidation comprises two separate 1-electron oxidations [38]. Electrooxidative dearomatization has proven to be an effective synthetic tool [39]. However, we have not found examples of electrochemical oxidation of PAPs applied in synthesis. Here, we report the synthesis of polycyclic aromatic quinones by anodic oxidation as a green alternative to our previous synthesis with SIBX [18].

## Results and Discussion

The electrochemical reactions were performed in the Flux module of the Syrris automated modular flow system [40] which provides a controlled geometry with a short distance between the electrodes, and easily reproduceable conditions. The electrochemical oxidation of phenols has been performed with platinum anodes [37,41], and carbon/platinum worked well for the oxidation of toluene dissolved in methanol with tetraethylammonium tosylate (Et_4_NOTs) as a supporting electrolyte within a flow system [32]. Et_4_NOTs is highly soluble in these solvents and can easily be removed by filtration through a pad of silia gel. Initially, we did a short screening of available electrode materials on the oxidation of commercially available 2-naphthol (**1a**, Table 1) obtaining the four-electron oxidation product 1,1-dimethoxynaphthalen-2(1*H*)-one (**2**). Best results were obtained with a carbon/platinum electrode pair, although stainless steel (SS) could also be used as cathode. The experiments were conducted with a 3:1 mixture of methanol/tetrahydrofuran (optimization not shown), where methanol further served as nucleophile. Some THF was needed to improve the solubility of some of the substrates. Acidic conditions (Table 1, entry 5) or methanol/water (Table 1, entry 6) gave a complex mixture with overoxidized products.

**Table 1:** Electrode and electrolyte effects on the electrochemical oxidation of 2-naphthol (**1a**).^a^

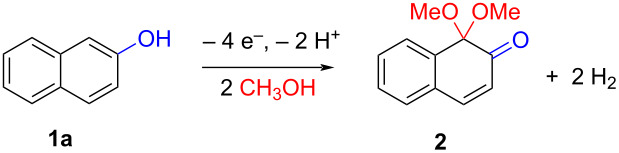				

Entry	Electrolyte	Anode/cathode^b^	Solvent^c^	Yield (%)^d^

1	Et_4_NOTS	C/C	MeOH/THF	0
2	Et_4_NOTS	C/SS	MeOH/THF	65
3	Et_4_NOTS	SS/C	MeOH/THF	0
4	Et_4_NOTS	C/Pt	MeOH/THF	84
5	Et_4_NOTS / TsOH	C/Pt	MeOH/THF	n/a^e^
6	Et_4_NOTS	C/Pt	MeOH/H_2_O	n/a^e^

^a^Experiments were conducted at room temperature with 0.01 M of **1a**, 0.05 M electrolyte, and 3.6 min residence time. ^b^Electrode materials: C: carbon filled PPS (polyphenylene sulfide), SS: stainless steel, Pt: platinum. ^c^Solvent ratio 3:1 (MeOH/THF) or 9:1 (MeOH/H_2_O). ^d^Isolated yield. ^e^Complex mixture/over-oxidation.

Although the desired oxidation is a 4-electon process, there will always be some extra current passing the cell that does not contribute to the reaction. Our optimization of the current on 1-chrysenol (**3b**) is given in Table 2. The reaction mixtures were introduced via a 10 mL-injection loop into the stream that was flowing through the Flux cell at a flowrate of 100 µL/min. The current was increased from 1 mA to 13 mA to increase the electron equivalents from 1 F/mol to 8 F/mol at a potential of 1.7–3.0 V. The crudes, after evaporation of the solvents, were further hydrolysed with a mixture of HCl, acetic acid, and water to release the quinones before purification. Further experimental details are given in Supporting Information File 1. The best result was obtained at 6 F/mol equivalents, giving 47% of quinone **4b** (Table 2, entry 4).

**Table 2:** Anodic methoxylation of 1-chrysenol (**3b**) at different electron equivalents.^a^

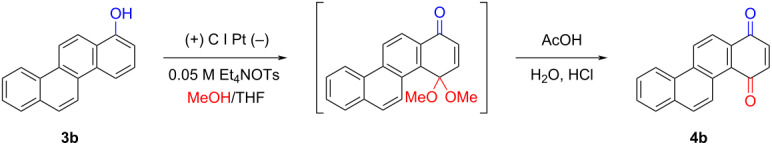			

Entry	Electrons (F/mol)	Current (mA)	Yield (%)^b^

1	1	2	33
2	2	3	31
3	4	6	40
4	6	9	47
5	7	11	42
6	8	13	34

^a^Conducted with 0.01 M substate and 100 µL/min flowrate (residence time 2.25 min). ^b^Isolated yield of chrysen-1,4-dione (**4b**).

These experiments, where the reaction mixture was passed through the cell a single time, gave rather low yields. This may be due to gas bubbles forming at the electrodes, disrupting the even distribution of electric current and potentially affecting the reaction [42–43]. When the size of a bubble is comparable to the width of a microchannel, the bubble tends to remain in the electrochemical cell and inhibits the reaction [44–45]. These gas slugs have been reported to block the ionic conduction path between electrodes and reduce the current down to 1/3 to 1/4 of its original value [43] and increase the activation overpotential of the cathode reaction [45].

To address these challenges in the single-pass operation, we directed our efforts toward recirculating the reaction mixture through the cell several times. This is often necessary to enhance the conversion of the electrochemical oxidation [46–47]. An increased electrolysis time is necessary as the conversion rate decreases significantly with the decay of the reactant concentration [46]. The reaction mixture was kept in a flask under stirring and pumped through the Flux cell and back to the flask. The flow rate was increased to 300 μL/min to faster flush out the evolved hydrogen gas from the cell. The Flux cell was operated in the galvanostatic mode at 9 mA until the substrate was consumed as monitored by TLC. Typically, the potential slowly increased from 1.7 V in the beginning of the experiments to approximately 2.9 V towards the end without any systematic variation between the compounds. The required residence time was mainly dependent on the conditions of the cell. It was building up in consecutive experiments but was reduced again by cleaning of the electrodes. The experiments collected in Table 3 and Table 4 typically had residence times between 3 and 7 minutes.

Substrates with the hydroxy group in the 2- or 3-position, i.e., **1a**, **3a**, **3c**, and **6a**, formed *o*-dimethoxylated products (Table 3). The oxidation of 2-naphthol (**1a**) to quinone acetal **2** by PhI(OAc)_2_ (PIDA) has been reported to provide yields ranging from 63% [48] to 76% [49], compared to 84% in our electrochemical oxidation that afforded 30 mg of **2** after 4 h (Table 3, entry 1). The dimethoxylated quinones are somewhat labile but can be purified by rapid silica gel chromatography and stored for a few weeks. The controlled current and anhydrous conditions helped avoiding overoxidation. The substrates leading to *p*-quinones were more prone to overoxidation. The electrochemical oxidation of phenanthren-4-ol (**6b**) provided the *p-*dimethoxylated product **8b** in 66% yield (87 mg), consuming 8 F/mol over a duration of 12.7 h (Table 3, entry 5). Next, quinone acetal **8b** was hydrolysed to phenanthrene-1,4-dione (**9b**) using aq acetic acid and HCl in 93% yield. The hydrolysis step went smoothly for all acetals. However, the methoxylated products from electrochemical oxidation of chrysen-1-ol (**3b)** and chrysen-6-ol (**3d**) rapidly hydrolysed to quinones during purification and could not be isolated. The attempted electrochemical oxidation of naphthalene-1-ol (**1b**) was unsuccessful; only small traces of multiple products were formed.

**Table 3:** Anodic methoxylation of PAPs followed by hydrolysis in two separate steps.^a^

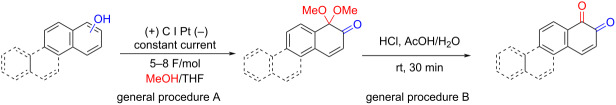			

Entry	PAP	Quinone acetal^b^	Quinone^c^

1	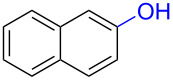 **1a**	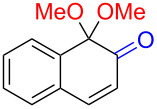 **2** (84%)	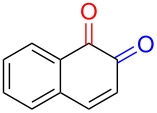 **5** (88%)
2	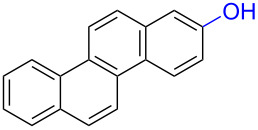 **3a**	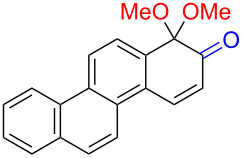 **7a** (72%)	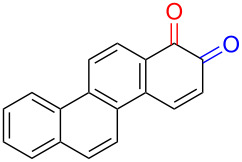 **4a** (69%)
3	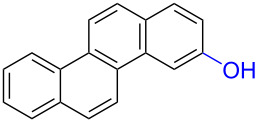 **3c**	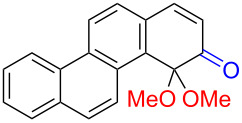 **7b** (79%)	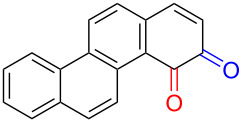 **4c** (96%)
4	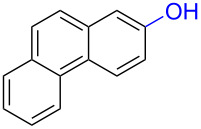 **6a**	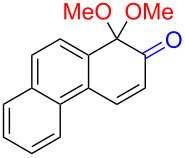 **8a** (57%)	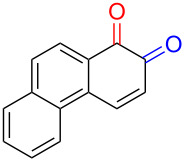 **9a** (90%)
5	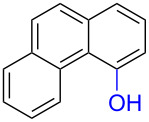 **6b**	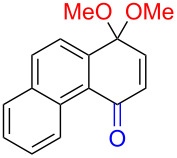 **8b** (66%)	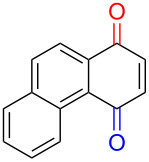 **9b** (93%)

^a^Reactions were carried out with 0.01 M substrate and 0.05 M of Et_4_NOTs in 3:1 MeOH/THF that was recirculated through the cell with 300 μL/min flow rate and 9 mA current. ^b^Isolated yields. ^c^Isolated yields calculated with the acetals as starting material.

The lability of the acetals prompted us to submit the crude intermediate directly to hydrolysis without prior isolation (Table 4). As mentioned above, having an aqueous reaction mixture in the electrochemical oxidation will give reduced yields. The overall yield of **9b** from **6b** while isolating the acetal in between steps (Table 3, entry 5) was 57%, whereas the direct hydrolysis of the crude provided an increased yield of 74% (Table 4, entry 7). Further, chrysene-1,4-dione (**4b**) and chrysene-5,6-dione (**4d**) were obtainable this way (Table 4, entries 3 and 5), while **1b** continued to be overoxidized and provided only traces of multiple products. Some overoxidation of **4d** was observed as 12-methoxychrysene-5,6-dione (**10**), but the alternative product chrysene-6,12-dione was not formed. In contrast to the *ortho*-selective oxidations of PAPs with SIBX [18], the electrochemical oxidation forms the *p*-quinones when possible. However, the *o*-quinones are formed in good yields from substrates where the *para*-position of the phenol is part of the further polycyclic aromatic skeleton. The products could be separated from the supporting electrolyte by dispersing the solids in ethyl acetate after removal of solvents from the reaction mixture. Thus, Et_4_NOTs can easily be recycled and reused for a greener reaction.

**Table 4:** Synthesis of *o*- and *p-*quinones without isolation of the acetal intermediate.^a^

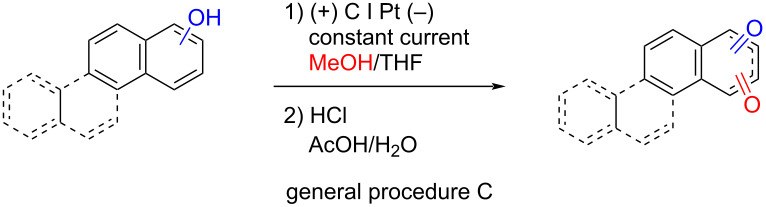		

Entry	PAP	Quinone^b^

1	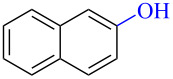 **1a**	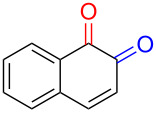 **5** (65%)
2	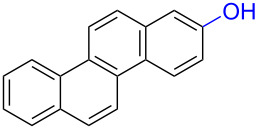 **3a**	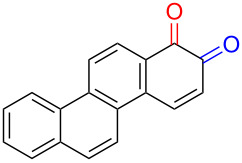 **4a** (63%)
3	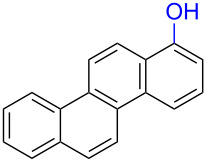 **3b**	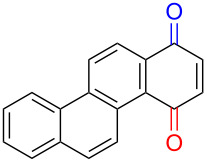 **4b** (65%)
4	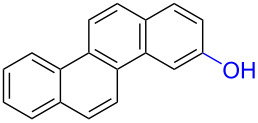 **3c**	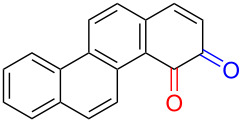 **4c** (58%)
5	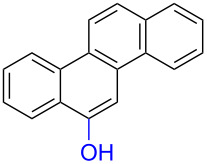 **3d**	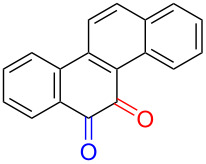 **4d** (72%)^c^
6	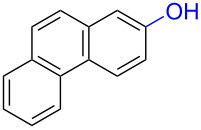 **6a**	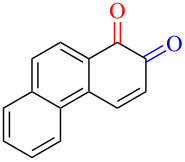 **9a** (49%)
7	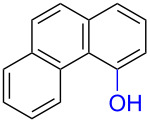 **6b**	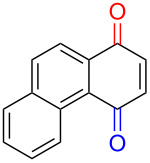 **9b** (74%)
8	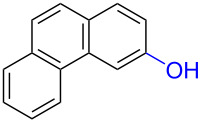 **6c**	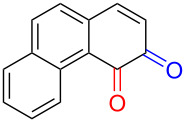 **9c** (81%)

^a^Reactions were carried out between 1.7 to 2.9 V with 0.01 M substrate and 0.05 M of Et_4_NOTs in 3:1 MeOH/THF that was recirculated through the cell with 300 μL/min flow rate and 9 mA current. ^b^Isolated yields. ^c^The further oxidized compound **10** (12-methoxychrysene-5,6-dione) was also isolated in 22% yield.

### Voltammetric studies

**Figure 1:**
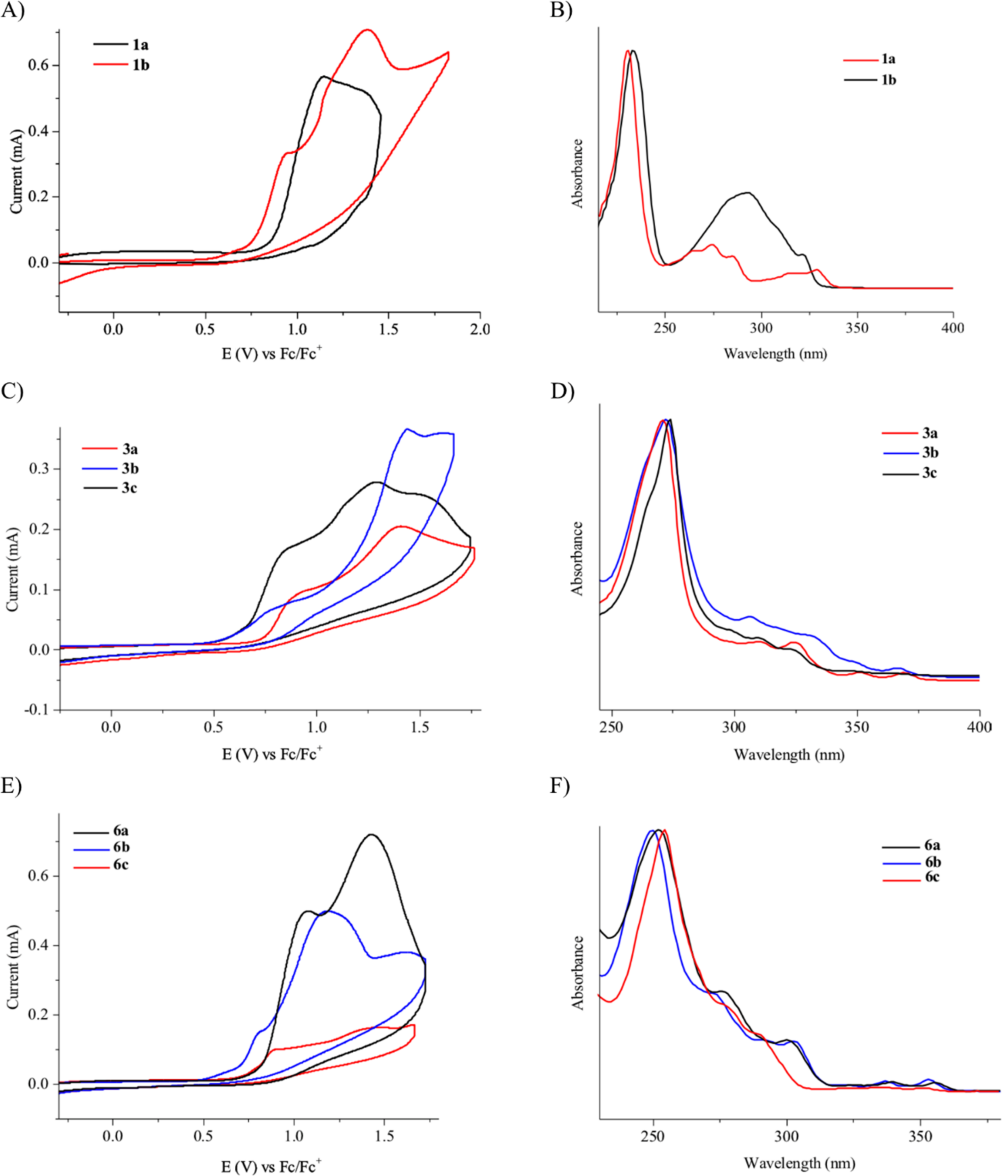
Cyclic voltammograms of PAPs first scan at 0.1 V/s in 0.1 M [NBu_4_] [PF_6_] in MeCN and UV–vis spectra of PAPs in DCM (≈10^−5^ M). A, B: naphthols **1a**,**b**. C, D: chrysenols **3a**–**c**. E, F: phenanthrols **6a–c**.

To investigate their redox behaviour, PAPs **1a**,**b** (Figure 1A), **3a–c** (Figure 1C), and **6a–c** (Figure 1E) were scanned between +2.2 V and −1.5 V. All compounds showed irreversible oxidation processes within the oxidative potential window, scaling from 0.79 V to 1.10 V vs Fe/Fe^+^. The oxidation peak potential difference between isomers of chrysenols **3** and phenanthrols **6** was 20–310 mV. No reduction peaks were observed in the reverse scan in solutions of neither chrysenols nor phenanthrols, suggesting a chemically irreversible reaction of the radical cation intermediate with the ensuing product no longer being electrochemically active within the potential window of the CV scans. However, a reduction peak was observed for compound **1b** (see Figure S2 in Supporting Information File 1). Naphthalene-1-ol (**1b**) gave a well-defined oxidation peak at 0.95 V (vs Fe/Fe^+^) while naphthalene-2-ol (**1a)** showed an oxidation peak at 1.14 V (vs Fe/Fe^+^). The oxidation peak potential difference between **1b** and **1a** was 190 mV.

PAHs undergo rapid irreversible chemical reactions upon electron transfer [50]. Unsubstituted PAHs display multielectron oxidation, but one-electron waves occur with electron-donating substituents in suitable positions. Panizza et al. [38] observed two one-electron oxidations in their cyclic voltammetry studies of **1a** in water and proposed the formation of a naphthyloxy radical and a naphthyloxy cation leading to the formation of **5**. Our CV studies exhibit oxidation peaks, which seem in line with what to expect for an electrochemical oxidation of PAPs.

Through the cyclic voltammetry experiments for the investigation of the redox behavior of the PAPs, an estimation of their highest occupied molecular orbital (HOMO) and lowest unoccupied molecular orbital (LUMO) energy levels can be derived via the oxidation onset potentials as shown in the literature [51]. The electrochemical properties of all products are summarized in Table 5. Individual CVs, with onset potentials indicated, are given in Supporting Information File 1. The optical properties of the PAPs were investigated by UV–vis absorption spectroscopy in 10^−5^ M solutions in CH_2_Cl_2_, as depicted in Figure 1. The UV–vis spectra of these compounds exhibited strong absorption in the region of 250–370 nm. These absorption bands are associated with π–π* and n–π* electronic transitions. The optical bandgap (*E*_g-opt_) values of the compounds determined from the absorption edge of the solution spectra are also summarized in Table 5. Although both HOMO and LUMO slightly vary between the compounds, the energy differences are quite the same for all compounds.

**Table 5:** Electrochemical properties of PAPs.

Compound	λ_max_^a^ (nm)	*E*_P1_\V _onset-ox_	*E*_ g-opt_ (eV)^b^	*E*_LUMO_ (eV)^c^	*E*_HOMO_ (eV)^d^

**1a**	231	0.87	3.66	−2.31	−5.97
**1b**	233	0.75	3.76	−2.09	−5.85
**3a**	271	0.74	3.30	−2.54	−5.84
**3b**	272	0.57	3.29	−2.38	−5.67
**3c**	274	0.64	3.28	−2.46	−5.74
**6a**	252	0.76	3.43	−2.43	−5.86
**6b**	250	0.70	3.42	−2.38	−5.80
**6c**	254	0.75	3.41	−2.44	−5.85

^a^Absorption maxima measured in DCM solutions at room temperature. ^b^The optical gap (*E*_g-opt_) was calculated from the onset point of the absorption spectra: *E*_g-opt_ = 1240/λ_onset_. ^c^HOMO energy calculated from the oxidation potential: *E*_HOMO_ = −(V_onset-ox_ + 5.1) eV. ^d^LUMO energy calculated from the difference between HOMO and optical gap (*E*_g-opt_).

**Scheme 3:**
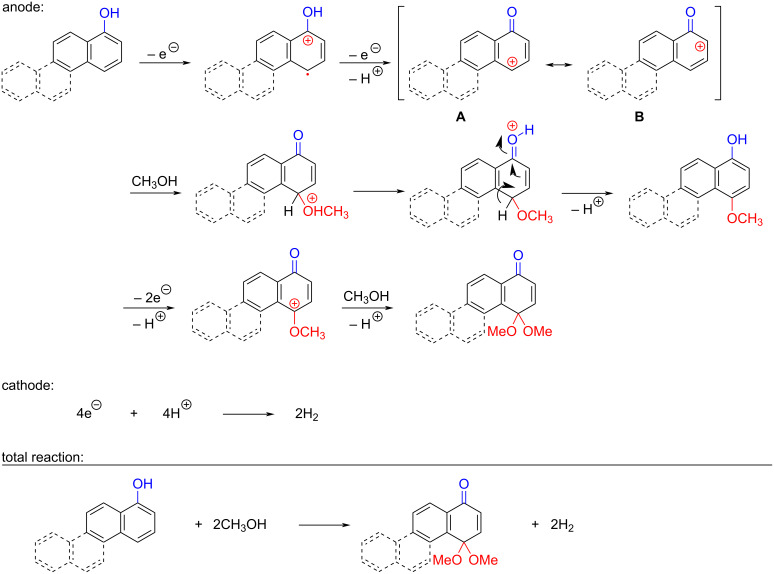
Proposed mechanism for the formation of *p*-dimethoxy acetals in the anodic oxidation of **1b** and **3b**.

The oxidation is initiated by an electron transfer from the substrate where the substrate will loose an electron more easily [42], and the free electron pairs of the hydroxy group are usually more difficult to ionize than π-electrons of the aromatic systems [52]. Studies by Swenton’s [41,53] and Barba’s [54] groups have established that a phenoxonium ion is formed, which is supported by further studies [37,39]. Based on this prior knowledge and our results, a mechanism for the anodic oxidation is proposed in Scheme 3. After two single-electron transfers [38], a cation is formed with two resonance structures (not counting further movement into the other aromatic rings destroying the aromaticity of one more ring). Resonance structure **A** has the cation in a benzylic position and will be the preferred site for nucleophilic attack of methanol compared to resonance structure **B**, which is further destabilized by the neighbouring ketone. A similar resonance hybrid will be formed for molecules substituted in the 4-position, like **6b**, explaining the selectivity towards *p*-quinones. Abstraction of a proton rearomatizes the molecule before another cation is formed in the following two one-electron oxidations. The abstracted protons are reduced to hydrogen gas at the cathode.

**Figure 2:**
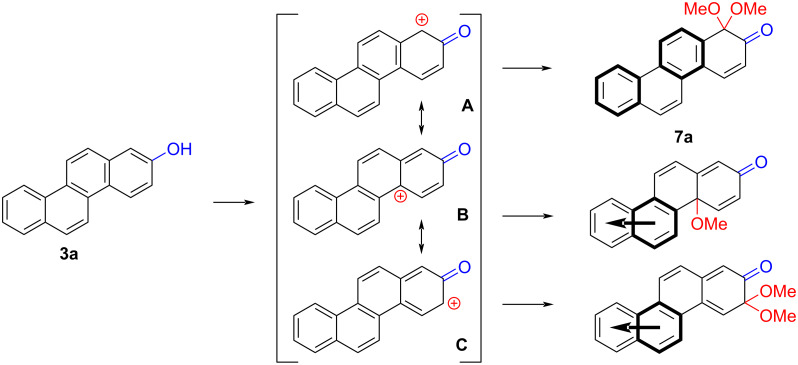
Resonance structures of the phenoxonium cation formed from 2-chrysenol (**3a**).

The formation of *o*-dimethoxy acetals and thus *o*-quinones can be considered through Clar’s aromatic sextet rules [55]. PAHs with more isolated and localized aromatic sextets are kinetically more stable than isomers with fewer aromatic π-sextets [56–57]. The relevant resonance structures of the phenoxonium ion of **3a**, and the Clar sextets of potential products are illustrated in Figure 2. The actual product, **7a**, has two isolated Clar sextets and should be favoured over the alternatives formed through cations **B** and **C** which have only one Clar sextet with two alternative positions.

## Conclusion

The electrochemical oxidation of polycyclic aromatic phenols to quinones represents a green alternative to chemical oxidants. Hydrogen gas evolution can be handled by recycling of the reaction mixture through the electrochemical flow cell to achieve high yields. Better yields are obtained with C/Pt electrode pair and methanol in the absence of water during the oxidation. The position of the hydroxy group controls the position of the quinone acetal to form a single product. *p*-Quinones are formed when the *para*-position to the hydroxy group is available for oxidation, while *o*-quinones are formed when the *para*-position is part of the conserved polyaromatic skeleton. All results are in accordance with an oxidation mechanism going through a phenoxonium cation.

## Experimental

The substrates **1a**,**b** and **6a**,**b** were obtained from our previous work [18]. Substrates **3a–c** and **6c** were synthesised by photochemical cyclisation of stilbenes [58], while **3d** was prepared according to literature [59]. The substrates were oxidized under galvanic (constant current) conditions in a Syrris Asia flow system with a 225 μL electrochemical flow cell equipped with a platinum-coated cathode and a carbon-filled PPS (polyphenylene sulfide) micro-channel anode separated by a polyetheretherketone (PEEK) gasket [40]. Further experimental details and characterization of new compounds are given in Supporting Information File 1.

### General procedure A: anodic oxidation with recirculating reaction solution

The reaction solution of 0.01 M PAPs and 0.05 M Et_4_NOTs was prepared by dissolving the chemicals in 3:1 MeOH/THF (10 mL). The reaction solution was circulated from a continuously stirred flask fitted with a slit septum, to the syringe pump, through the Flux cell, and back at 300 µL/min flow rate. The target current was set at 9 mA and when the voltage exceeded 3.2 V, the reaction would be stopped to avoid over-oxidation. The reaction was monitored by TLC until the substrate was consumed. After completed reaction, the system was flushed with methanol to collect all reaction mixture. The solvents were evaporated under reduced pressure, and the crude purified by column chromatography to isolate the product.

### General procedure B: hydrolysis of acetals

To a solution of the quinone acetal (0.15 mmol) in acetic acid (4 mL) were added 2 drops of conc. HCl and 3–4 drops of water. The mixture was stirred at room temperature for 0.5 h and poured into ice water (5 mL). The precipitated quinone was filtered off, thoroughly washed with water, and dried under vacuum to yield the pure product.

### General procedure C: combined electrochemical oxidation and hydrolysis

Following general procedure A, the reaction solution with the PAP was recirculated at 300 µL/min flow rate through the Flux Cell with 9 mA electrical current until the substrate was consumed. Solvents were removed under reduced pressure and the crude dispersed in ethyl acetate (3 × 3 mL) and filtered to remove the electrolyte. The filtrate was concentrated under reduced pressure and the crude dissolved in acetic acid (3 mL) before hydrolysis according to general procedure B.

### Voltammetric studies

Voltammetric experiments were carried out using a Princeton Applied Research versaSTAT 3 potentiostat, connected to a three-electrode setup using Pt wires as working and pseudo reference electrodes and Pt mesh as counter electrode. The experimental conditions for the cyclic voltammetry (CV) scans were kept constant at 0.1 V/s. Voltammetric studies were conducted in 0.1 M tetrabutylammonium hexafluorophosphate ([NBu_4_] [PF_6_]) solution in acetonitrile. The solvent was dried and degassed using N_2_ prior to each experiment. All experiments were conducted at room temperature. All redox potentials were calibrated against ferrocene/ferrocenium (Fc/Fc^+^) redox couple.

## Supporting Information

File 1 Detailed experimental procedures and characterization data of new molecules together with individual cyclic voltagrams with onset potentials.

## Data Availability

All data that supports the findings of this study is available in the published article and/or the supporting information to this article.
