# Supplementary material for: Synthesis of polycyclic aromatic quinones by continuous flow electrochemical oxidation: anodic methoxylation of polycyclic aromatic phenols (PAPs)
Source: Beilstein J Org Chem. 2024 Jul 24;20:1746–57. doi: 10.3762/bjoc.20.153 (PMC11285069; doi:10.3762/bjoc.20.153)
Supplement: File 1 — Detailed experimental procedures and characterization data of new molecules together with individual cyclic voltagrams with onset potentials. [file Beilstein_J_Org_Chem-20-1746-s001.pdf]

BEILSTEIN JOURNAL OF ORGANIC CHEMISTRY

Supporting Information

for

Synthesis of polycyclic aromatic quinones by continuous flow electrochemical oxidation: anodic methoxylation of polycyclic aromatic phenols (PAPs)

Hiwot M. Tiruye, Solon Economopoulos and Kåre B. Jørgensen

Beilstein J. Org. Chem. **2024,** *20,* 1746–1757. doi:10.3762/bjoc.20.153

Detailed experimental procedures and characterization data of new molecules together with individual cyclic voltagrams with onset potentials

License and Terms: This is a supporting information file under the terms of the Creative Commons Attribution License (https://creativecommons.org/licenses/by/4.0). Please note that the reuse, redistribution and reproduction in particular requires that the author(s) and source are credited and that individual graphics may be subject to special legal provisions.
The license is subject to the *Beilstein Journal of Organic Chemistry* terms and conditions: (https://www.beilstein-journals.org/bjoc/terms)

Table of contents

1. Electrochemical equipment S2
2. Experimental procedure for single-pass experiments (Table 2) S3
3. Cyclic voltammetry measurements S4
4. Electrochemical synthesis S6
    4.1. General information S6
    4.2. Characterization of compounds S8
5. ^{1}H NMR and $^{13}C\{^{1}H\}$ NMR S16
    1,1-Dimethoxynaphthalen-2(1H)-one (**2**) S17
    Chrysene-1,4-dione (**4b**) S18
    1,1-Dimethoxychrysen-2(1H)-one (**7a**) S19
    4,4-Dimethoxychrysen-3(4H)-one (**7b**) S20
    1,1-dimethoxyphenanthren-2(1H)-one (**8a**) S21
    1,1-Dimethoxy phenanthrene-4(1H)-one (**8b**) S22
    Phenanthren-1,4-dione (**9b**) S23
    12-Methoxychrysene-5,6-dione (**10**) S24
6. References S26

1. Electrochemical equipment

For all electrochemical reactions, a Syrris Asia modular flow system was used. All solvents were purchased HPLC-grade since only pure solvent flows through the cell. Continuous-flow electrochemical oxidation was performed using the Asia Flux Module. The Flux cell was constructed from two rectangular electrode plates (5.3 × 4.0 cm). The design provided a channel depth of approximately 0.2 mm (with the spacer fitted into the recessed channel of the electrode in the compressed cell) and a channel width of 1.5 mm, giving a cell volume of 0.225 mL. Parameters used to perform oxidation reactions were constant current (galvanostatic) at the power output (mA), constant voltage (V), the concentration of the solution applied to the cell (Mol/L), and the flow rate of the solution through the cell (mL/min).

The Flux module is controlled by constant current or voltage modus controlling the electrochemical cell. Unless otherwise specified we applied a platinum coated cathode and a carbon filled PPS (polyphenylene sulfide) micro-channel anode separated by a polyetheretherketone (PEEK) gasket. The reaction mixture is pumped through the system via a pulse-free syringe pump.

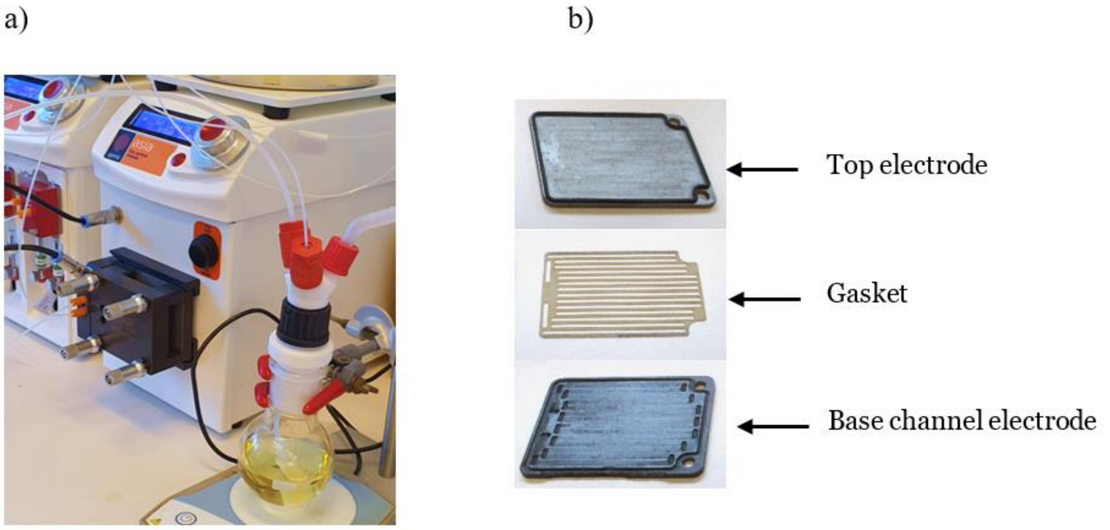

Figure S1. Experimental setup: a) The reaction mixture is circulated from the reaction flask on the right, through the pump and the Syrris Asia Flux module and back to the reaction flask; b) The cell was assembled with a carbon channel electrode, a PEEK gasket and a platinum top electrode.

2. *Experimental procedure for single-pass experiments (Table 2)*

A 0.01 M solution of chrysen-1-ol (**3b**) was prepared from an electrolyte solution of 10 mL (0.05M Et_4NOTs in MeOH:THF 3:1). The Flux module was assembled using a Pt-cathode and a C-anode. The mixture was loaded into an injection loop from which it was injected into the flow of pure electrolyte solution at 100 µL/min flow rate. Accordingly, the current was increased from 1 mA to 13 mA to achieve increasing of electron equivalents from 1 F/mol to 8 F/ mol (Table S1). The colour of the reaction mixture changed from colourless at the input to yellow at the output. In each experiment the plug of 10 mL of product solution was collected. Solvents were evaporated, the crude dissolved in ethyl acetate (3 × 3 mL) and filtered to remove the electrolyte. The solvents were removed in vacuo and the remains subjected to the next reaction without further purification. Acetic acid (3 mL), 3–4 drops of conc. HCl and 3–4 drops of water were added to the remains and the mixture was stirred at room temperature for 30 min. It was then poured into ice water (5 mL) and filtered to obtain the quinone as a pure solid.

Table S1. Sequence of electrochemical oxidation at constant flow rate.

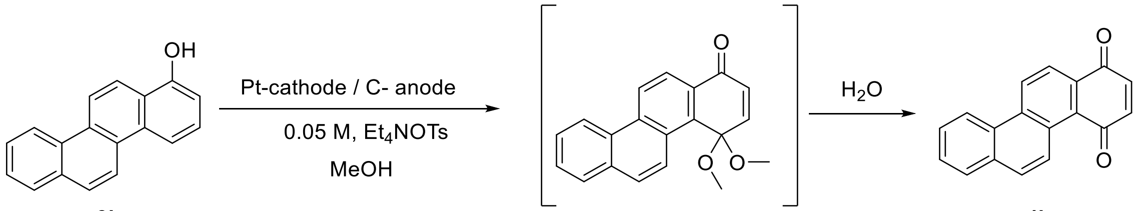

Exp	Electrons (F/mol)	Current (mA)	Voltage (V)	Wt. (mg)	Yield (%)[b]
1	1	2	1.7	8.4	33
2	2	3	1.9	8.0	31
3	4	6	2.2	10.2	40
4	6	9	2.6	12.2	47
5	7	11	2.9	10.8	42
6	8	13	3.0	8.9	34

[a] Flow rates of 100 µL/min (2.25 min residence time) and 0.01 M of chrysen-1-ol (**3b**).[b] Isolated yield of **4b**.

3. *Cyclic voltammetry measurements*

Voltammetric experiments were carried out using a Princeton Applied Research versaSTAT 3 potentiostat, connected to a three-electrode setup using Pt wires as working and pseudo reference electrodes and Pt mesh as counter electrode. The experimental conditions for the cyclic voltammetry (CV) scans were kept constant at 0.1 V s^{-1}. Voltammetric studies were conducted in 0.1 M tetrabutylammonium hexafluorophosphate ([NBu_4] [PF_6]), solution in acetonitrile. The solvent was dried and degassed using N_2 prior to each experiment. All experiments were conducted at room temperature. All redox potentials were calibrated against ferrocene/ferrocenium (Fc/Fc^+) redox couple. Samples were prepared with a substrate in 3 mL of 0.1M tetra-*n*-butylammonium hexafluorophosphate in dry, degassed acetonitrile. The cyclic voltammetry was carried out in a platinum wire (working electrode, counter electrode, and reference electrode) and a scan rate of 100 mV s^{-1}. The obtained value was referenced to Fc/Fc^+. The cyclic voltammetry graphs with onset potentials indicated are given in Figures S2–S4.

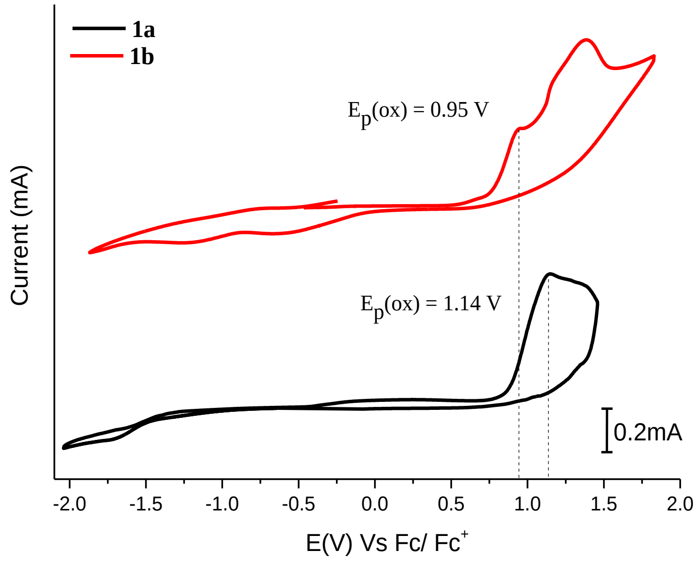

Figure S2. Cyclic voltammograms of naphthol isomers **1a,b** (MeCN, 0.1 M [NBu_4] [PF_6]). CV: v = 0.1 Vs^{-1}.

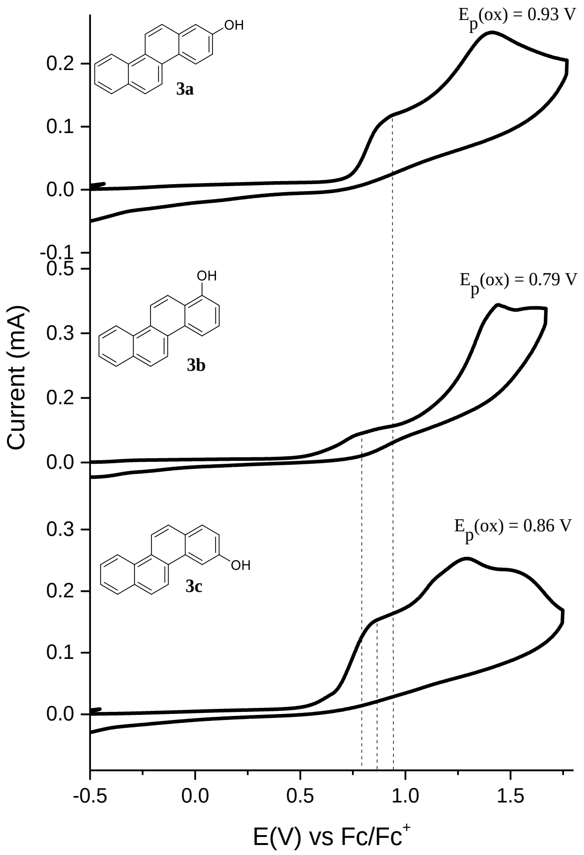

Figure S3. Cyclic voltammograms of chrysenol isomers **3a–c** (MeCN, 0.1 M [NBu_4] [PF_6]). CV: v = 0.1 Vs^{-1}.

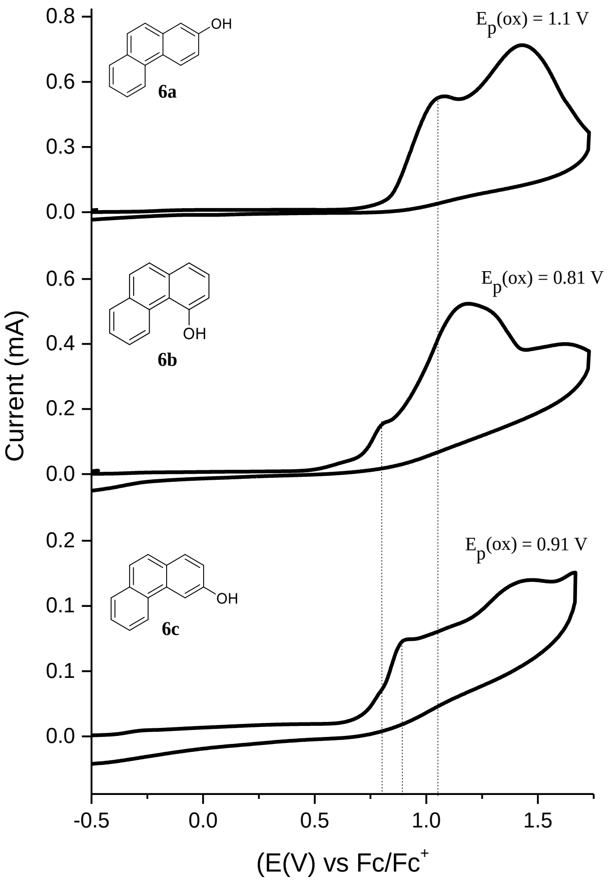

Figure S4. Cyclic voltammograms of phenanthrol isomers **6a–c** (MeCN, 0.1 M [NBu_4] [PF_6]). CV: $v = 0.1\ Vs^{-1}$.

4. Electrochemical synthesis

4.1.General information

All solvents were purchased HPLC-grade and used without further purification. 2-Phenanthrol (**6a**) and 4-phenanthrol (**6b**) were obtained from our previous work.[1] 3-Phenanthrol (**6c**), 1-, 2-, 3-, and 6-chrysenol (**3a–d**) were synthesized according to literature.[2,3] Naphthols were commercially available. The reactions were monitored by thin-layer chromatography using commercial F_{254} silica-gel plates or by GC–MS (CP-Sil 8 CB, 50 m length and 0.25 mm diameter). ^{1}H NMR, and $^{13}C\{^{1}H\}$ NMR spectra were recorded on a 400 MHz Bruker AVANCE III spectrometer. Chemical shifts (δ) are given in ppm relative to the TMS signal (0.00 ppm) for ^{1}H NMR and the solvent signal for ^{13}C NMR ($CDCl_3$ at 77.0 ppm and DMSO-d_6 at 39.52 ppm) with coupling constants in Hz. The structure of the products was verified by 2D NMR. Ultraviolet–visible (UV–vis) absorption spectra were recorded on a VWR UV-1600PC

spectrophotometer. Melting points were measured on a Büchi MP-3 melting point apparatus (uncorrected). Infrared (IR) spectra were measured as neat compounds on an Agilent Carey 630 FTIR spectrophotometer equipped with an attenuated total reflectance (ATR) sampling module. Absorption strength is designated as vs (very strong), s (strong), m (medium), and w (weak). HRMS were measured on an Orbitrap Exploris 120 with an APCI probe by Thermo Fischer Scientific; or a JMS T100 GCAccuTOFTM EI-TOF from JEOL.

The electrochemical oxidations were conducted on the Syrris® Asia flow-system under constant current (galvanostatic) conditions in their Flux Module equipped with a 225 µL flow cell, assembled with a platinum-coated cathode and a carbon-filled PPS (polyphenylene sulfide) micro-channel anode separated by a polyetheretherketone (PEEK) gasket making an approx. 70 cm long channel. Reaction flow was controlled using the Syrris Syringe Pump (SyrDos 2.5 mL syringes).

General procedure A: Anodic oxidation with recirculating reaction solution

The reaction solution of 0.01 M PAPs and 0.05 M Et_4NOTs was prepared by dissolving the chemicals in 3:1 MeOH:THF (10 mL). The reaction solution was circulated from a continuously stirred flask fitted with a slit septum, to the Syringe pump, through the Flux cell and back at 300 µL/min flow rate. The target current was set at 9 mA and when the voltage exceeded 3.2 V, the reaction would be stopped to avoid over-oxidation. The reaction was monitored by TLC until the substrate was consumed. After completed reaction, the system was flushed with methanol to collect all reaction mixture. The solvents were evaporated under reduced pressure, and the crude purified by column chromatography to isolate the product.

General procedure B: hydrolysis of acetals

To a solution of quinone acetal (0.15 mmol) in acetic acid (4 mL) were added 2 drops of conc. HCl and 3–4 drops of water. The mixture was stirred at room temperature for 0.5 h and poured into ice water (5 mL). The precipitated quinone was filtered off, thoroughly washed with water, and dried under vacuum to yield the pure product.

General procedure C: combined electrochemical oxidation and hydrolysis

Following general procedure A, the reaction solution with the PAP was recirculated at 300 µL/min flow rate through the Flux Cell with 9 mA electrical current until the substrate was consumed. Solvents were removed under reduced pressure and the crude dispersed in ethyl acetate (3 × 3 mL) and filtered to remove the electrolyte. The filtrate was concentrated under reduced pressure and the crude dissolved in acetic acid (3 mL) before hydrolysis according to general procedure B.

4.2. Characterization of compounds

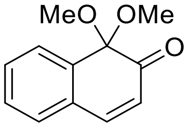

1,1-Dimethoxynaphthalen-2(1H)-one (**2**):

Following general procedure A, a 0.01 M solution of **1a** (30 mg, 0.21 mmol) and Et_4NOTs (0.32 g, 0.05 M) in 3:1 MeOH:THF (21 mL) was recirculated through the Flux Cell for 4 h to afford **2** (35.7 mg, 84%) as a yellow oil. NMR data were in agreement with those previously reported.[4]

^{1}H NMR (400 MHz, $CDCl_3$): δ = 3.28 (s, 6 H), 6.12 (d, *J* = 10.0 Hz, 1H), 7.31 (m, 2H), 7.40 (td, *J* = 7.4 Hz, 1.4 Hz, 1H), 7.45 (td, *J* = 7.5, 1.4 Hz, 1H), 7.71 (dd, *J* = 7.4, 1.0 Hz, 1H).

^{13}C{^{1}H} NMR (100 MHz, $CDCl_3$): δ = 51.9, 95.5, .4, 125.2, 128.2, 129.6, 129.8, 130.1, 131.6, 137.8, 144.2, 195.6 ppm.

IR(ATR): 2939 (w), 2833 (w), 1672 (m), 1628 (w), 1600 (w), 1451(w), 1205 (W)1073 (s), 993 (m), 812 (m), 757 (m) cm^{-1}.

HRMS (ESI) *m/z*: calcd for $C_{12}H_{12}O_3$, 227.0679 [M + H]$^+$; found, 227.0678.

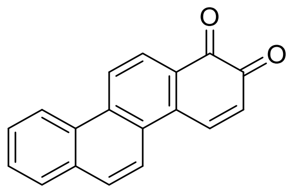

Chrysen-1,2-dione (**4a**):

Following general procedure B, to a solution of **7a** (36 mg, 0.12 mmol) in acetic acid (3 mL) were added 2 drops of conc. HCl and 3–4 drops of water to afford **4a** (21.3 mg, 69%) as a brown solid. Mp. 237–239 °C (dec.) (Lit.[5] 250-251 °C (dec.)). NMR data were identical to those values reported in our previous work.[1]

Following general procedure C, a solution of **3a** (30 mg, 0.12 mmol) and Et_4NOTs (0.18 g, 0.05 M) in 3:1 methanol:THF (12 mL) was recirculated through the Flux Cell for 6 h and hydrolysed to afford **4a** (19.7 mg, 63%) as a brown solid.

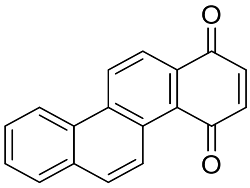

Chrysene-1,4-dione (**4b**):

Following general procedure C, a solution of **3b** (40 mg, 0.16 mmol) and Et_4NOTs (0.25 g, 0.05 M) in 3:1 MeOH:THF (16 mL) was recirculated through the Flux Cell for 6 h and hydrolysed to afford **4b** (27.0 mg, 65%) as a golden-bronze chrystal. Mp. 196– 197 ^{o}C (Heptane) (lit [5] 206.5-207.5).

^{1}H NMR (400 MHz, $CDCl_3$): δ = 6.97 (d, *J* = 10.1 Hz, 1H), 7.01 (d, *J* = 10.2 Hz, 1H), 7.70-7.73 (m, 2H), 7.92-7.94 (m, 2H), 7.98 (d, *J* = 9.4 Hz, 1H), 8.34 (d, *J* = 8.6 Hz, 1H), 8.69-8.72 (m, 1H), 9.06 (d, *J* = 8.7, 1H), 9.45 (d, *J* = 9.6, 1H).

^{13}C{^{1}H} NMR (100 MHz, $CDCl_3$): δ = 121.8, 123.39, 123.4, 124.4, 127.6, 128.6, 128.7, 128.9, 129.6, 130.1, 131.6, 132.1, 132.4, 134.9, 136.1, 141.2, 185.8, 188.4 ppm.

IR(ATR): 3052 (w), 2922 (m), 2853 (w), 1725 (m), 1650 (s), 1613 (m), 1580 (m), 1528 (m), 1491 (m), 1414 (m), 1303 (s), 1232 (m), 1072 (m), 1040 (m), 840 (m), 826 (m), 806 (m), 768 (m), 749 (s), 704 (m) cm^{-1}.

HRMS (ESI) *m/z*: calcd for $C_{18}H_{10}O_2$, 259.0754 $[M + H]^{+}$; found, 259.0753.

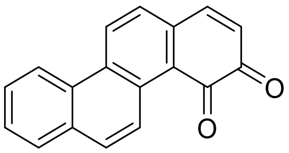

Chrysen-3,4-dione (**4c**):

Following general procedure B, to a solution of **7b** (30 mg, 0.10 mmol) in acetic acid (3 mL) were added 2 drops of conc. HCl and 3–4 drops of water to afford **4c** (24.3 mg, 96%) red solid. NMR data agree with values reported in our previous work.[1]

Following general procedure C, a 0.01 M solution of **3c** (20 mg, 0.08 mmol) and Et_4NOTs (0.08 g, 0.05 M) in 3:1 MeOH:THF (8 mL) was recirculated through the Flux Cell for 4 h and hydrolysed to afford **4c** (12.0 mg, 58%) as a red solid. NMR data were identical to those values reported in our previous work.[1]

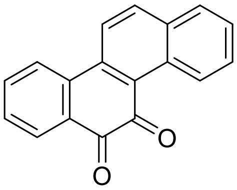

Crysene-5,6-dione (**4d**):

Following general procedure C, a solution of **3d** (65 mg, 0.27 mmol) and Et_4NOTs (0.40 g, 0.05 M) in MeOH:THF 3:1 (27 mL) was recirculated through the cell for 6 h and hydrolysed to afford a crude mixture of **9d** and **10**. The crude was separated by flash chromatography to afford **4d** (49.9 mg, 72%) as an orange solid, **10** (17.0 mg, 22%) as red crystal. NMR data of **4d** were identical to those values reported in our previous work.[1]

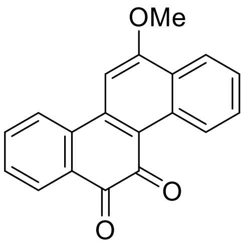

12-Methoxychrysene-5,6-dione (**10**):

^{1}H NMR (400 MHz, $CDCl_3$): δ = 4.23 (s, 3H), 7.38 (s, 1H), 7.50 (t, *J* = 7.5 Hz, 1.0 Hz, 1H), 7.55 (t, *J* = 7.6 Hz, 1.2 Hz, 1H), 7.70- 7.78 (m, *J* = 2H), 8.05 (d, *J* = 8.1 Hz, 1H), 8.16 (dd, *J* = 7.7, 1.2 Hz, 1H), 8.29 (d, *J* = 8.4 Hz, 1H), 9.50 (d, *J* = 8.5, 1H).

IR(ATR): 3066 (w), 2928 (m), 2855 (w), 1724 (w), 1665 (s), 1578 (s), 1489 (m), 1375 (m), 1345 (m), 1203 (w), 1073 (s), 957 (m), 845 (m), 755 (s), 724 (w) cm^{-1}.

HRMS (EI) *m/z*: calcd for $C_{19}H_{12}O_3$, 311.0684 $[M + Na]^+$; found, 311.0683.

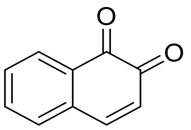

Naphthalen-1,2-dione (**5**):

Following general procedure B, to a solution of 1,1-dimethoxynaphthalen-2(1*H*)-one (**2**, 30 mg, 0.15 mmol) in acetic acid (3 mL) were added 2 drops of conc. HCl and 3–4 drops of water to yield **5** (24.0 mg, 88%) as a red solid. Mp. 121- 124 °C (EtOAc). (lit.[6] 126-129 °C, lit.[7] 144-146 °C) NMR data were in agreement with those previously reported. [1, 7]

Following general procedure C, a 0.01 M solution of **1a** (30 mg, 0.21 mmol) and Et_4NOTs (0.31 g, 0.05 M) in 3:1 MeOH:THF (21 mL) was recirculated (100 µL/min) through the Flux Cell (3 mA) for 5 h and hydrolysed to afford **5** (21.5 mg, 65%) as a red solid. NMR data were identical to those values reported in our previous work.[1]

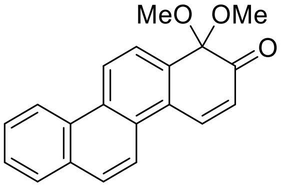

1,1-Dimethoxychrysen-2(1H)-one (**7a**):

Following general procedure A, a 0.01 M solution of **3a** (50 mg, 0.21 mmol) and Et_4NOTs (0.31 g, 0.05 M) in 3:1 MeOH:THF (21 mL) was recirculated through the cell for 6 h to afford **7a** (46.3 mg, 72%) as a yellow solid. Mp. 156 °C

^{1}H NMR (400 MHz, $CDCl_3$): δ = 3.33 (s, 6H), 6.34 (d, *J* = 10.6 Hz, 1H), 7.65-7.74 (m, 2H), 7.90 (d, *J* = 9.3 Hz, 1H), 7.94 (dd, *J* = 7.7, 1.3 Hz, 1H), 8.03 (d, *J* = 8.6, 1H), 8.16 (d, *J* = 9.2 Hz, 1H), 8.31 (d, *J* = 10.4 Hz, 1H), 8.72 (d, *J* = 8.2 Hz, 1H), 8.85 (d, *J*=8.7 Hz, 1H).

^{13}C{^{1}H} NMR (100 MHz, $CDCl_3$): δ = 52.1. 53.6, 95.5, 120.3, 123.1, 125.2, 125.3, 125.6, 127.3, 127.4, 127.6, 128.7, 129.0, 129.4, 130.2, 131.6, 131.8, 137.8, 139.0, 195.7 ppm.

IR(ATR): 3058 (w), 2922 (m), 2852 (w), 1676 (s), 1597 (w), 1450 (m), 1277 (m), 1259 (m), 1193 (m), 1107 (m), 1061 (s), 971 (m), 815 (s), 759 (s), 698 (w) cm^{-1}.

HRMS (ESI) *m/z*: calcd for $C_{20}H_{16}O_3$, 327.0991 [M + Na]$^+$; found, 327.0992.

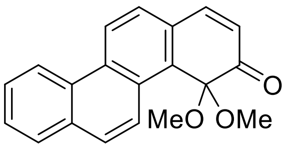

4,4-Dimethoxychrysen-3(4h)-one (**7b**):

Following general procedure A, a 0.01 M solution of **3c** (80 mg, 0.33 mmol) and Et_4NOTs (0.49 g, 0.05 M) in 3:1 MeOH:THF (33 mL) was recirculated through the cell for 8 h to afford **7b** (78.5 mg, 79%) as yellow needle formed crystals. Mp. 148-151 °C (Heptane).

^1H NMR (400 MHz, $CDCl_3$): δ = 3.26 (s, 6H), 6.35 (d, *J* = 9.9 Hz, 1H), 7.56 (d, *J* = 9.4 Hz, 2H), 7.64-7.68 (m, 2H), 7.82 (d, *J* = 9.4 Hz, 1H), 7.92 (dd, *J* = 7.80, 2.39 Hz, 1H), 8.69 (dd, *J* = 9.1, 2.0 Hz, 1H), 8.80 (d, *J* = 8.4 Hz, 1H), 8.87 (d, *J* = 9.5 Hz, 1H).

$^{13}C\{^1H\}$ NMR (100 MHz, $CDCl_3$): δ = 51.2, 96.3, 123.0, 124.8, 125.0, 125.4, 126.8, 127.2, 127.6, 128.1, 128.5, 130.0, 130.4, 131.6, 132.1, 132.5, 134.5, 146.2, 195.6 ppm.

IR(ATR): 3045 (w), 2924 (m), 2853 (w), 1724 (m), 1665 (s), 1623 (w), 1490 (m), 1232 (m), 1142 (m), 1063 (s), 1029 (m), 995 (m), 955 (m), 844 (m), 830 (m), 751 (s), 700 (w) cm^{-1};

HRMS (ESI) *m/z*: calcd for $C_{20}H_{16}O_3$, 327.0991 $[M + H]^+$; found, 327.0991.

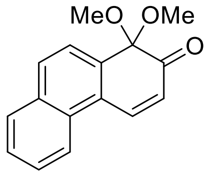

1,1-Dimethoxyphenanthren-2(1H)-one (**8a**):

Following general procedure A, a 0.01 M solution of **6a** (50 mg, 0.26 mmol) and Et_4NOTs (0.39 g, 0.05 M) in 3:1 MeOH:THF (26 mL) was recirculated through the Flux Cell for 7 h to afford **8a** (37.5 mg, 57%) as a yellow solid. Mp. 113-114 °C.

^1H NMR (400 MHz, $CDCl_3$): δ = 3.30 (s, 6 H), 6.32 (d, *J* = 10.4 Hz, 1H), 7.56-7.65 (m, 2H), 7.86 (d, *J* = 8.6 Hz, 1H), 7.90 (d, *J* = 8.1 Hz, 1H), 7.98 (d, *J* = 8.5 Hz, 1H,) 8.23 (d, *J* = 8.2 Hz, 1H,), 8.24 (d, *J* = 10.4 Hz, 1H,).

$^{13}C\{^1H\}$ NMR (100 MHz, $CDCl_3$): δ = 51.8, 95.2, 122.1, 124.6, 125.0, 126.8, 126.9, 127.6, 128.9, 130.3, 131.0, 134.0, 137.8, 138.6, 195.7 ppm.

IR(ATR): 2927 (w), 2855 (w), 1678 (m), 1463 (w), 1047 (vs), 963 (m), 818 (m), 789 (m), 682 (w) cm^{-1}.

HRMS (ESI) *m/z*: calcd for $C_{16}H_{14}O_3Na$, 277.0841 $[M + Na]^+$; found, 277.0835.

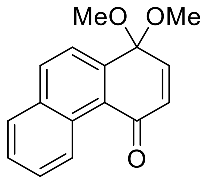

1,1-Dimethoxyphenanthren-4(1H)-one (**8b**):

Following general procedure A, a 0.01 M solution of **6b** (100 mg, 0.52 mmol) and Et_4NOTs (0.77 mg, 0.05 M) in 3:1 MeOH:THF (52 mL) was recirculated through the cell for 12 h to afford **8b** (87.0 mg, 66%) as a yellow solid.

^{1}H NMR (400 MHz, $CDCl_3$): δ = 3.19 (s, 6 H), 6.66 (d, *J* = 10.5 Hz, 1H), 6.85 (d, *J* = 10.2 Hz, 1H), 7.59 (t, *J* = 7.5 Hz, 1.1 Hz, 1H), 7.70 (t, *J* = 7.8 Hz, 1.5 Hz 1H), 7.84 (d, *J* = 8.6 Hz, 1H,) 7.88 (d, *J* = 8.2 Hz, 1H,), 8.13 (d, *J* = 8.6 Hz, 1H,), 9.64 (d, *J* = 8.5 Hz, 1H,).

^{13}C{^{1}H} NMR (100 MHz, $CDCl_3$): δ = 51.4, 96.0, 123.4, 126.7, 127.1, 127.2, 128.5, 129.4, 130.5, 134.0, 135.0, 135.2, 141.1, 142.4, 186.6 ppm.

IR(ATR): IR(ATR): 2983 (w), 1735 (s), 1593 (w), 1455 (m), 1372 (m), 1227 (vs), 1119 (m), 1033 (vs), 1010 (m), 731 (m), 630 (m) cm^{-1}.

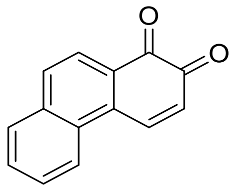

Phenanthrene-1,2-dione (**9a**):

Following general procedure B, to a solution of **8a** (12 mg, 0.05 mmol) in acetic acid (1 mL) were added 2 drops of conc. HCl and 1–2 drops of water to afford **9a** (9.4 mg, 90%) as a red needle formed crystals. NMR data were identical to those values reported in our previous work.[1]

Following general procedure C, a solution of **6a** (50 mg, 0.26 mmol) and electrolyte (0.39 mg, 0.05 M) in 3:1 MeOH:THF (26 mL) was recirculated through the Flux Cell for 7 h and hydrolysed to afford **9a** (26.5 mg, 49%) as a red solid.

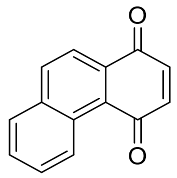

Phenanthrene-1,4-dione (**9b**):

Following general procedure B, to a solution of **8b** (82 mg, 0.31 mmol) in acetic acid (6 mL) were added 4 drops of conc. HCl and 8 drops of water to afford **9b** (62.8 mg, 93%) as a yellow needle. m.p. 149- 150 °C (EtOAc). (lit.[8] 151–152 °C). NMR data were in agreement with those previously reported.[8]

Following general procedure C, a solution of **6b** (15 mg, 0.08 mmol) and Et_4NOTs (0.12 g, 0.05 M) in 3:1 MeOH:THF (8 mL) was recirculated through the Flux Cell for 2 h and hydrolysed to afford **9b** (11.8 mg, 74%) as a yellow needles.

^1H NMR (400 MHz, $CDCl_3$): δ = 6.97 (dd, *J* = 14.6, 10.2 Hz, 2H), 7.66 (t, *J* = 7.5 Hz, 1H), 7.75 (t, *J* = 8.5 Hz, 1H), 7.91 (d, *J* = 8.1 Hz, 1H), 8.18 (dd, *J* = 10.0, 8.8 Hz, 2H,), 9.56 (d, *J* = 8.8 Hz, 1H).

$^{13}C\{^1H\}$ NMR (100 MHz, $CDCl_3$): δ = 121.9, 127.2, 127.9, 128.7, 130.0, 130.2, 132.3, 135.2, 135.8, 136.6, 140.6, 186.0, 188.3 ppm.

IR(ATR): 3058 (w), 2957 (w), 2925 (m), 2856 (w), 1725 (m), 1650 (s), 1615 (m), 1585 (m), 1457 (m), 1432 (m), 1377 (m), 1301 (s), 1154 (w), 1071 (s), 961 (w), 832 (s), 811 (s), 754 (s), 688 (m) cm^{-1}.

HRMS (ESI) *m/z*: calcd for $C_{14}H_8O_2$, 209.0597 $[M + H]^+$; found, 209.0597.

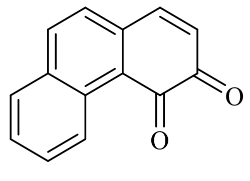

4.3.8. *Phenanthren-3,4-dione* (**9c**).

Following general procedure C, a solution of **6c** (20 mg, 0.10 mmol) and Et_4NOTs (0.16 g, 0.05 M) in 3:1 MeOH:THF (10 mL) was recirculated through the cell for 8.5 h to yield **9c** (16.9 mg, 81%) as a brown solid. NMR data were identical to those values reported in our previous work.[1]

5. ^{1}H NMR and ^{13}C{^{1}H} NMR

Chloroform-impurities:

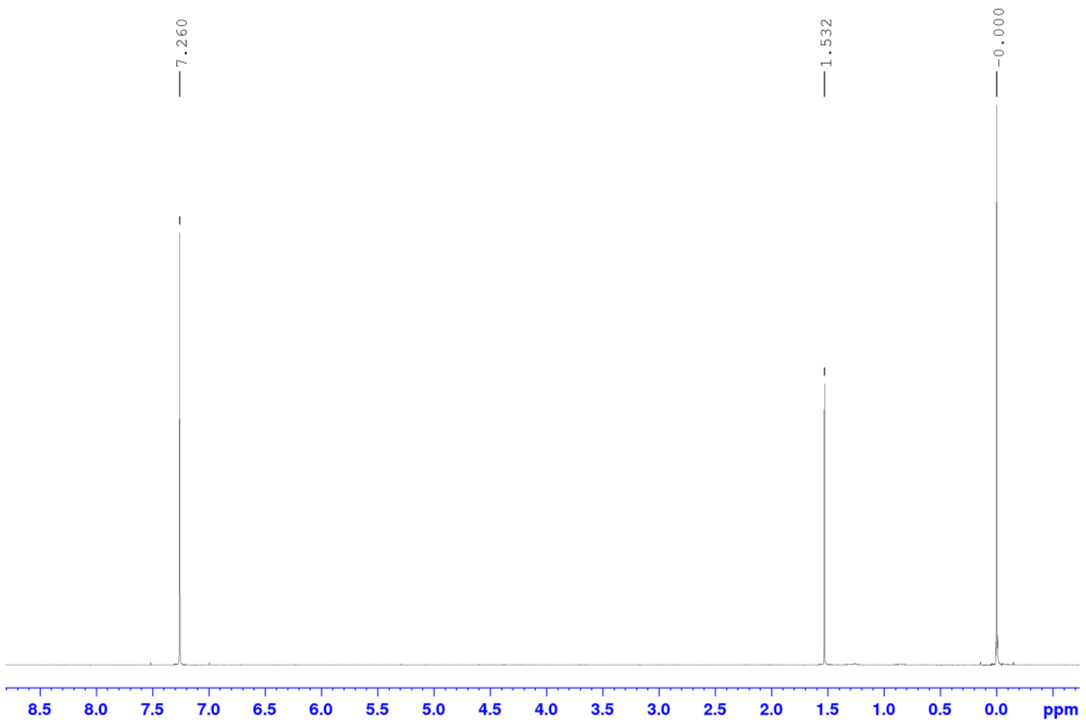

1,1-Dimethoxynaphthalen-2(1H)-one (**2**)

^{1}H NMR (400 MHz, $CDCl_3$)

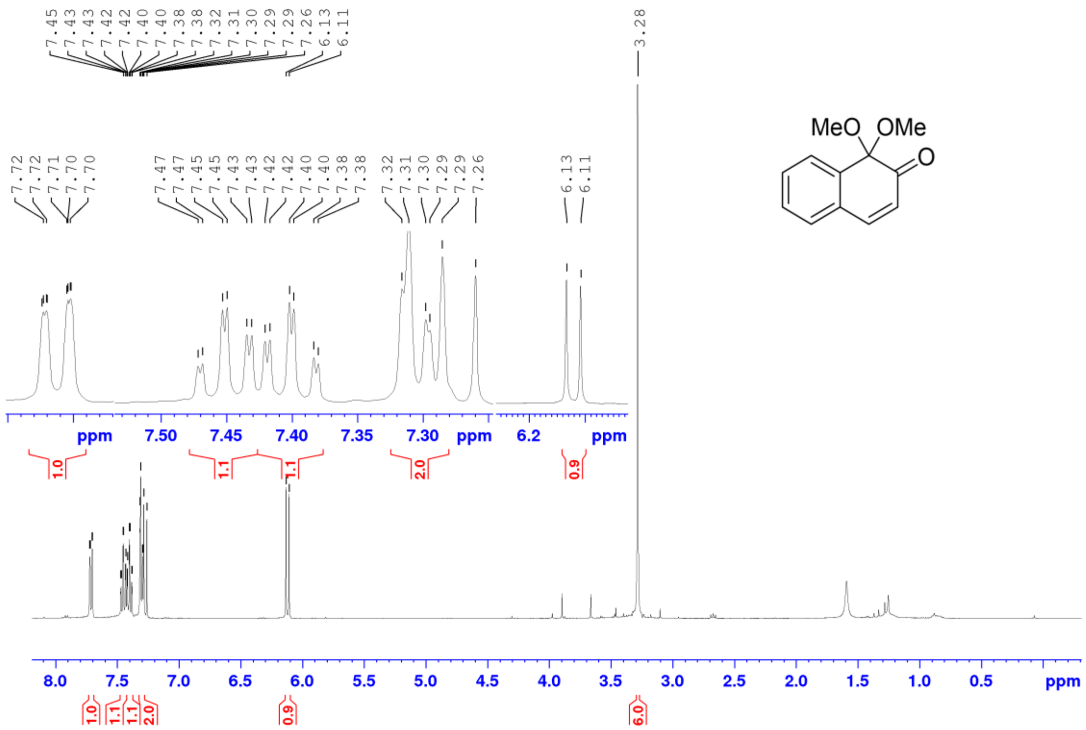

$^{13}C\{^{1}H\}$ NMR (100 MHz, $CDCl_3$)

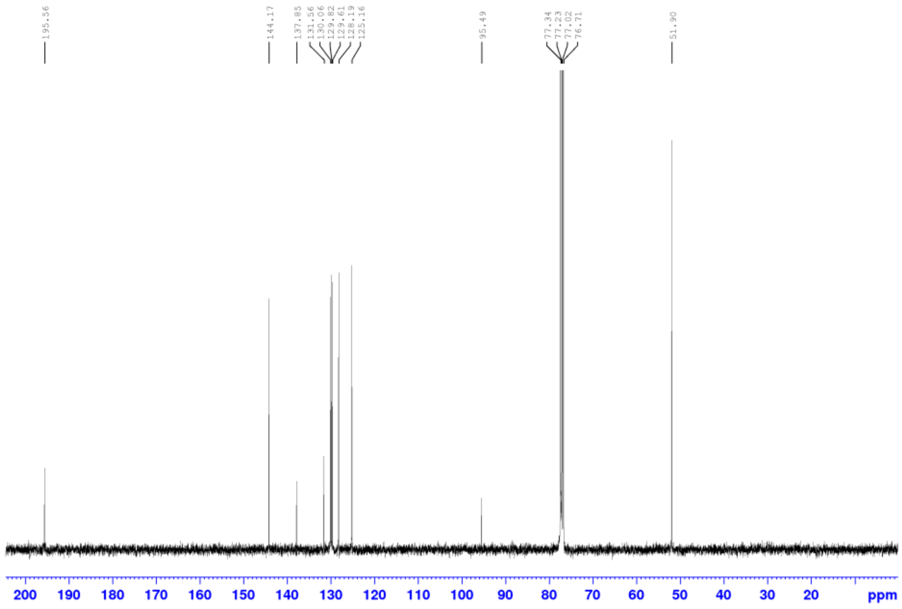

*Chrysene-1,4-dione (**4b**)*

^{1}H NMR (400 MHz, $CDCl_3$)

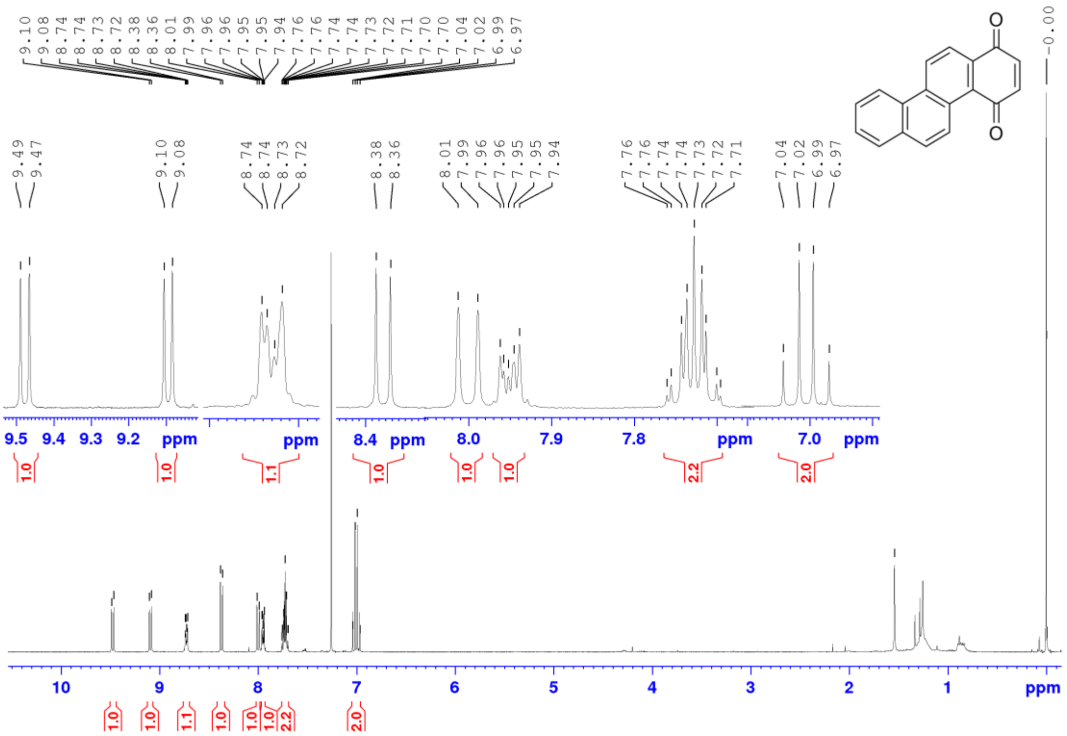

^{13}C{^{1}H} NMR (100 MHz, $CDCl_3$)

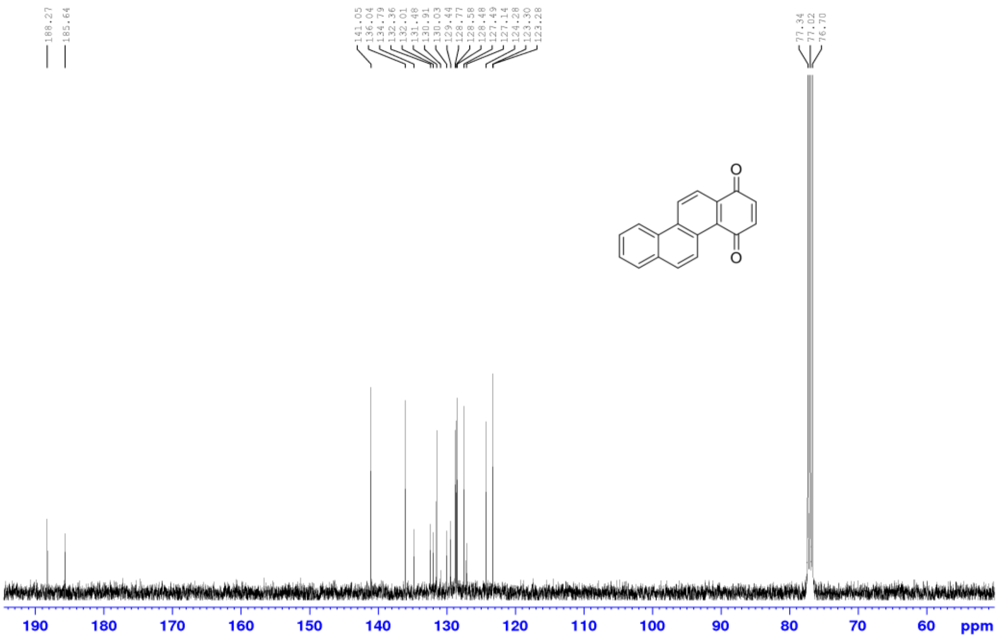

*1,1-Dimethoxychrysen-2(1H)-one (**7a**)*

^{1}H NMR (400 MHz, $CDCl_3$)

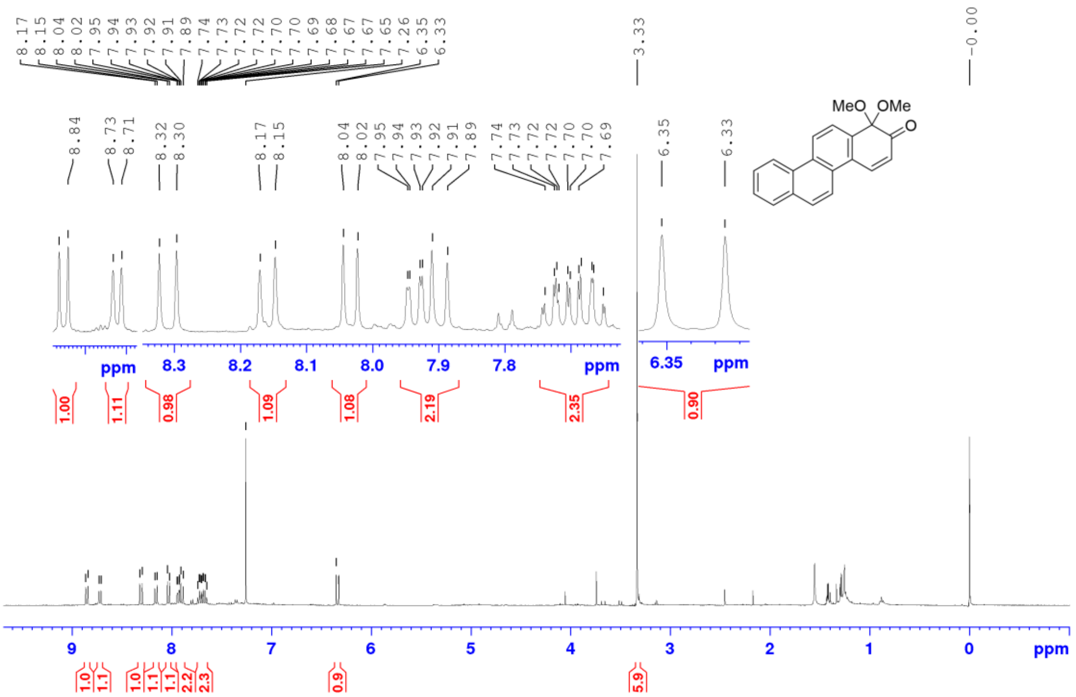

^{13}C{^{1}H} NMR (100 MHz, $CDCl_3$)

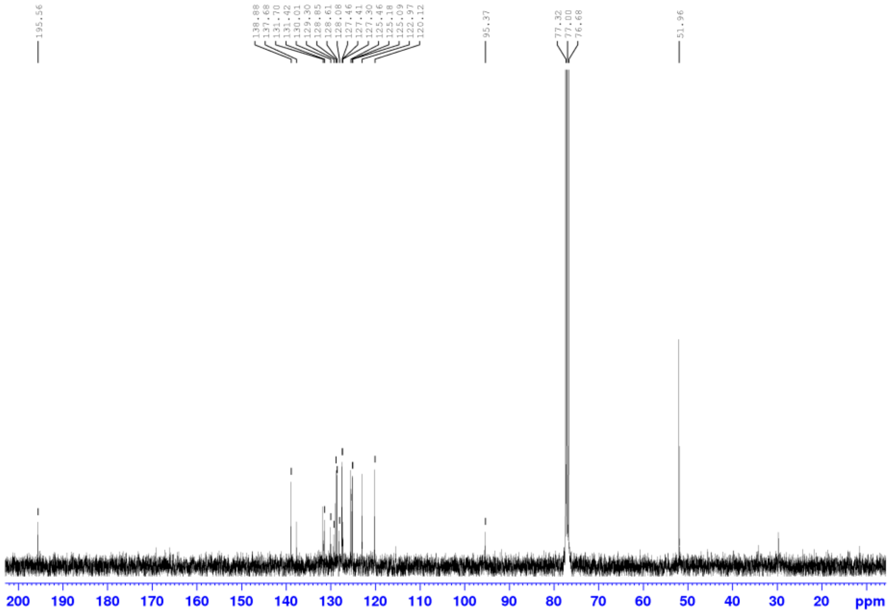

*4,4-Dimethoxychrysen-3(4H)-one (**7b**)*

^{1}H NMR (400 MHz, $CDCl_3$)

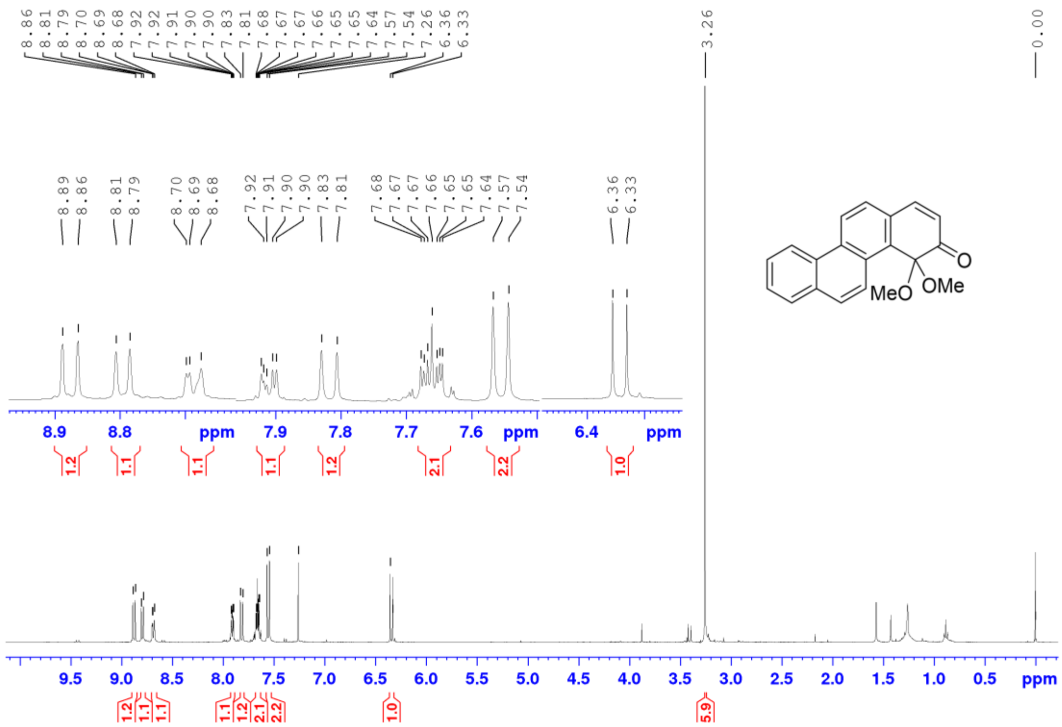

$^{13}C\{^{1}H\}$ NMR (100 MHz, $CDCl_3$)

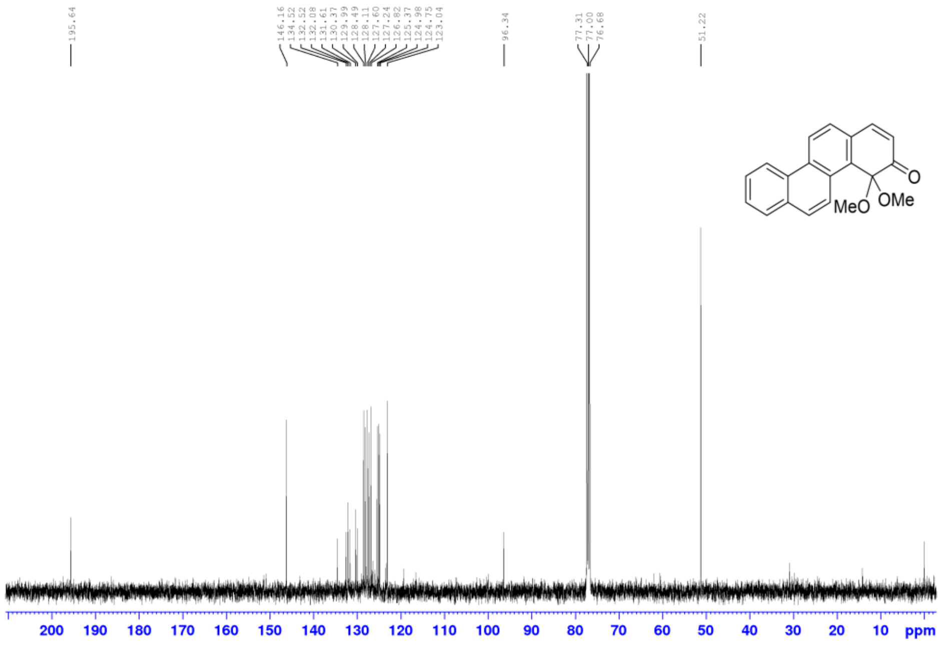

*1,1-Dimethoxyphenanthren-2(1*H*)-one (**8a**)*

^{1}H NMR (400 MHz, $CDCl_3$)

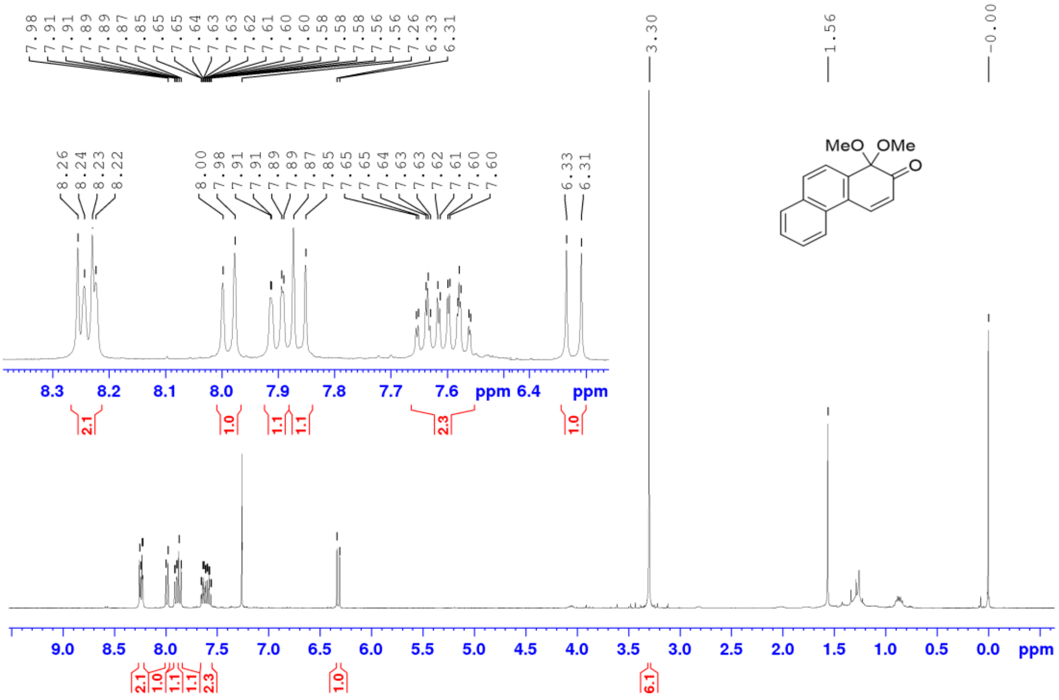

^{13}C{^{1}H} NMR (100 MHz, $CDCl_3$)

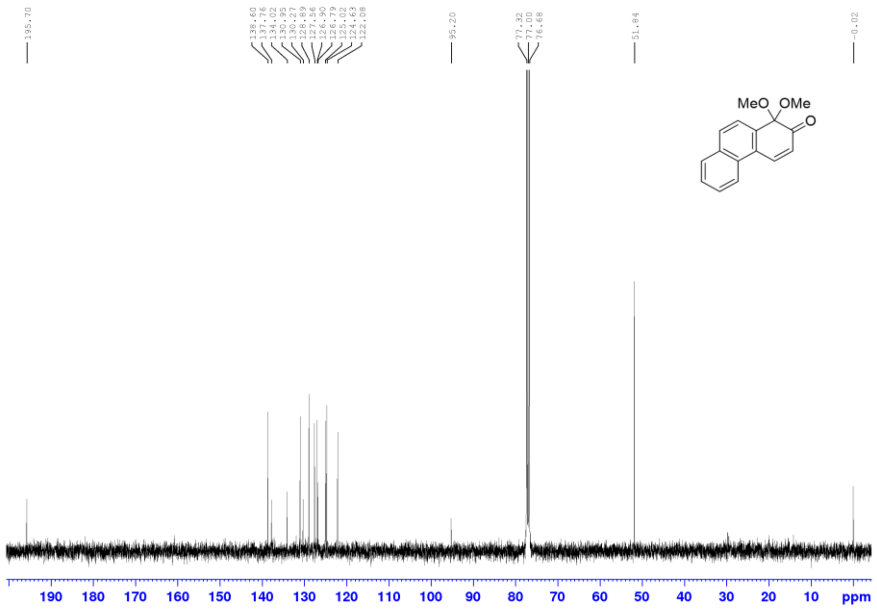

*1,1-Dimethoxy phenanthrene-4(1H)-one (**8b**)*

^{1}H NMR (400 MHz, $CDCl_3$)

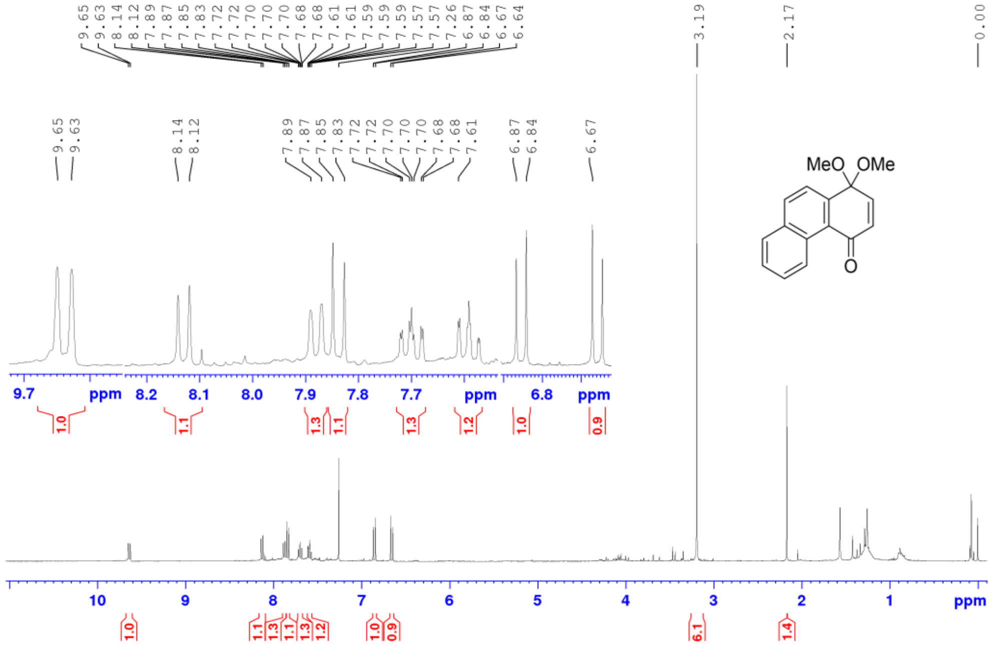

$^{13}C\{^{1}H\}$ NMR (100 MHz, $CDCl_3$)

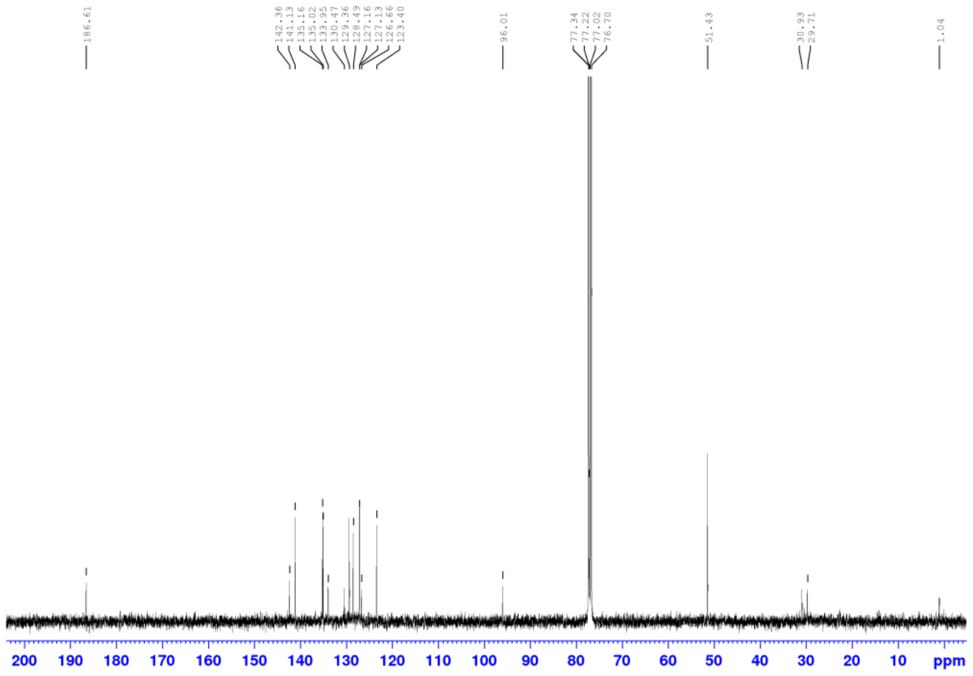

*Phenanthren-1,4-dione (**9b**)*

^{1}H NMR (400 MHz, $CDCl_3$)

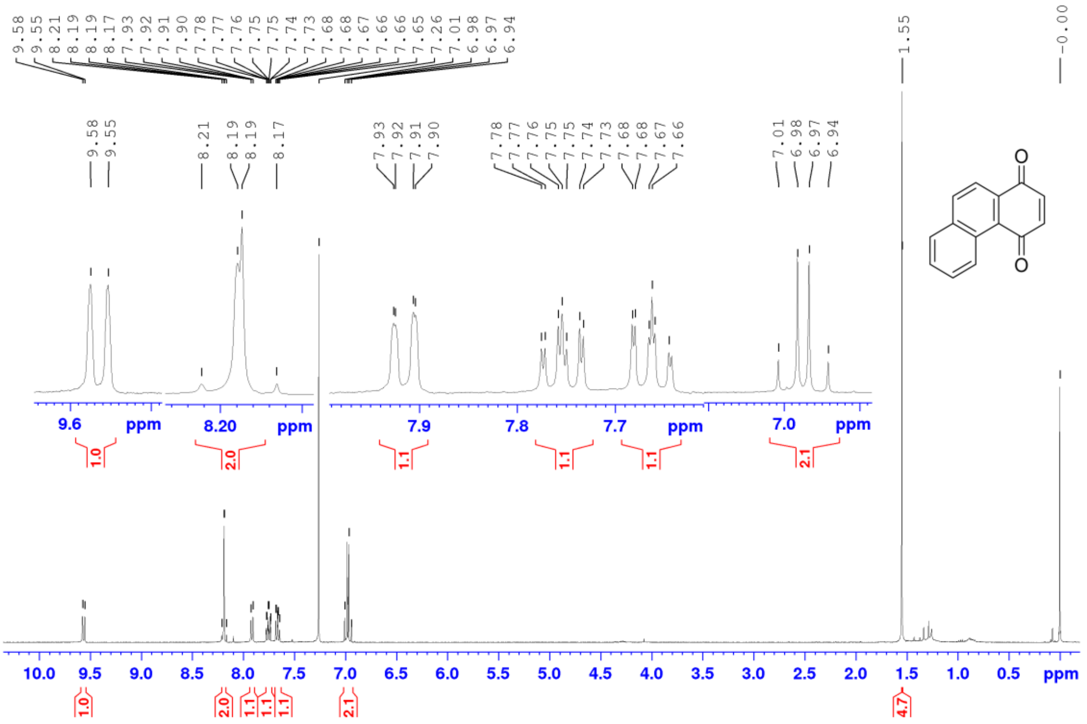

$^{13}C\{^{1}H\}$ NMR (100 MHz, $CDCl_3$)

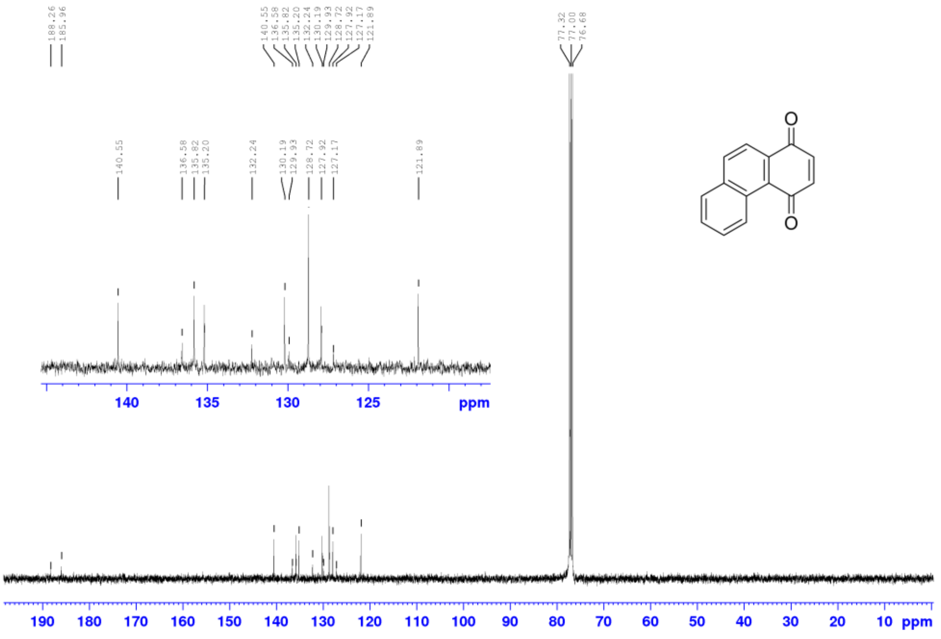

*12-Methoxychrysene-5,6-dione (**10**)*

^{1}H NMR (400 MHz, $CDCl_3$)

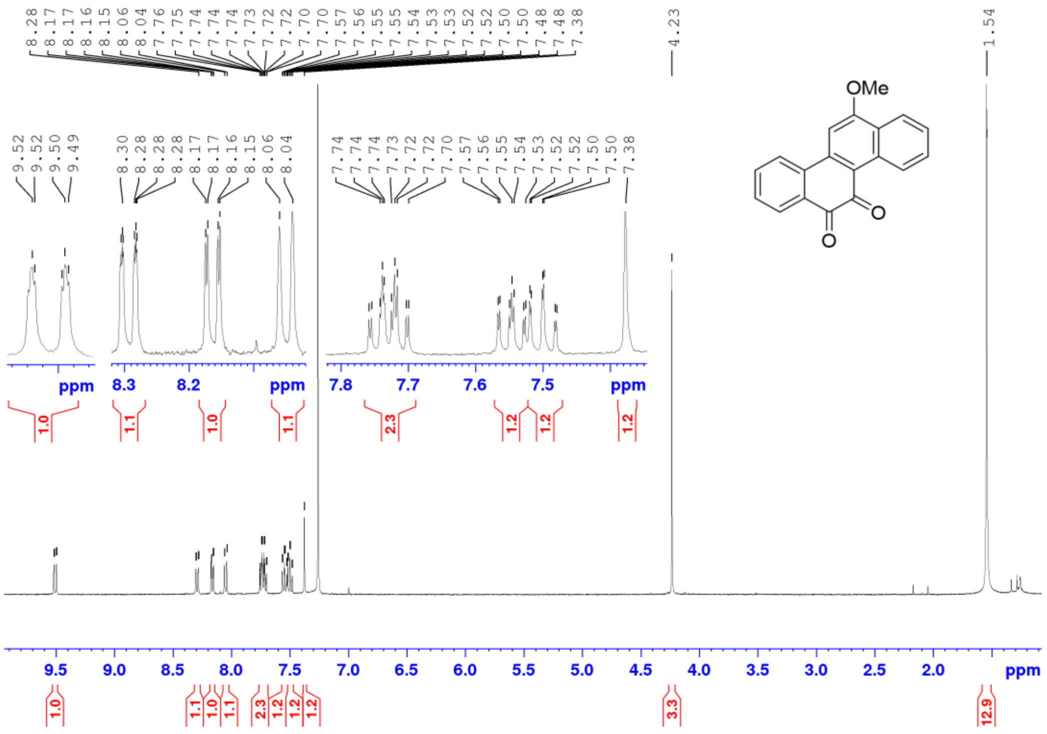

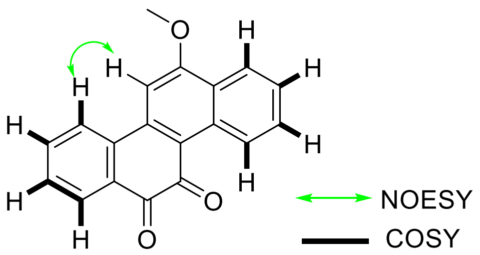
O
H
NOESY
COSY

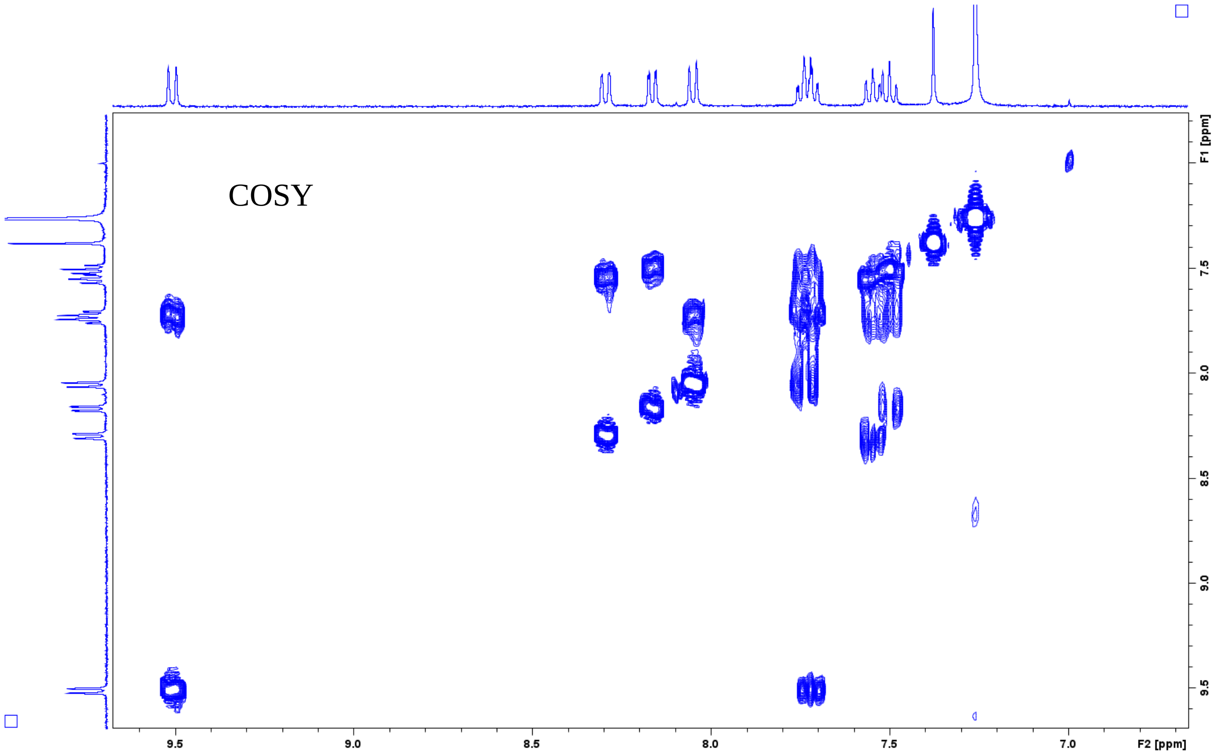
COSY
F1 [ppm]
F2 [ppm]
9.5
9.0
8.5
8.0
7.5
7.0

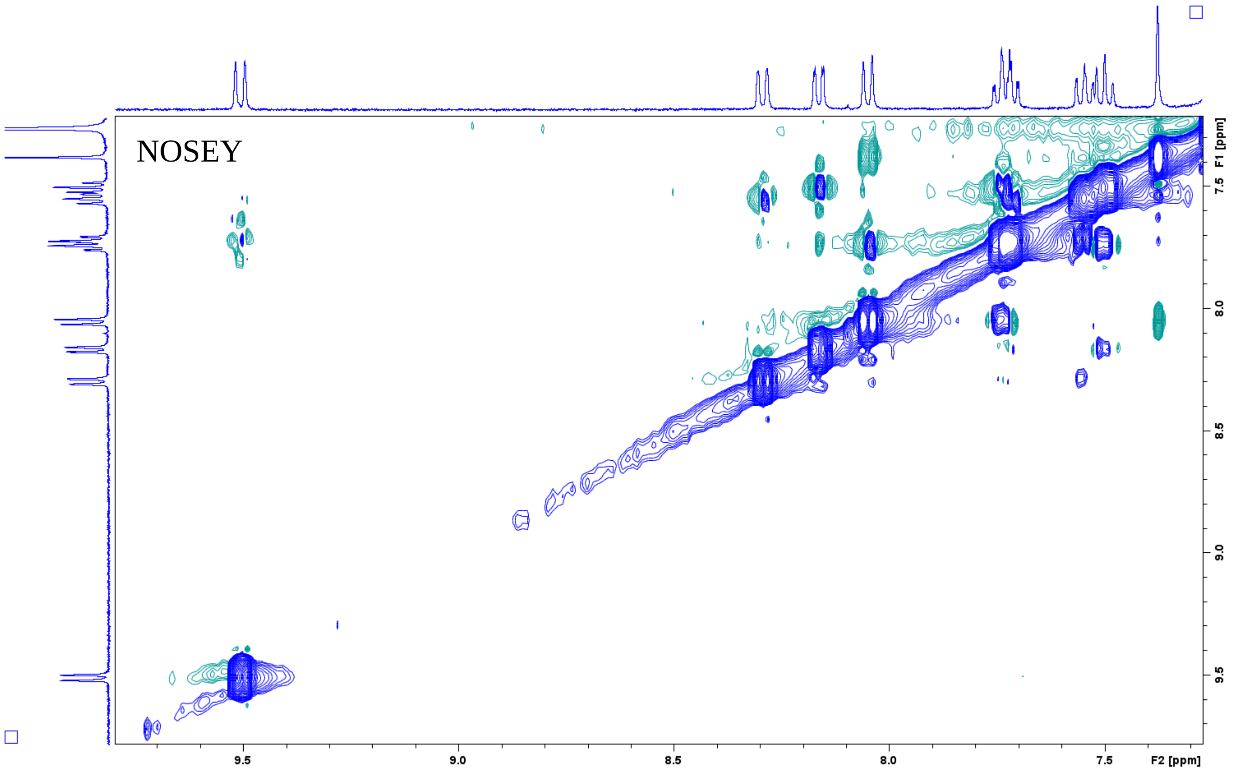
NOSEY
F1 [ppm]
F2 [ppm]
9.5
9.0
8.5
8.0
7.5

6. References

(1) Tiruye, H. M.; Jørgensen, K. B. Oxidative synthesis of ortho-quinones from hydroxy-PAHs by stabilized formulation of 2-iodoxybenzoic acid (SIBX). *Tetrahedron* **2022**, *129*. DOI: 10.1016/j.tet.2022.133144.
(2) Jørgensen, K. B.; Joensen, M. Photochemical Synthesis of Chrysenols. *Polycyclic Aromatic Compounds* **2008**, *28* (4-5), 362-372. DOI: 10.1080/10406630802374580.
(3) Matsushima, T.; Kobayashi, S.; Watanabe, S. Air-Driven Potassium Iodide-Mediated Oxidative Photocyclization of Stilbene Derivatives. *J Org. Chem.* **2016**, *81* (17), 7799-7806. DOI: 10.1021/acs.joc.6b01450.
(4) Mascall, K. C.; Jacobi, P. A. Furanosteroid studies. Improved synthesis of the A,B,C,E-ring core of viridin. *Tetrahedron Lett.* **2012**, *53* (13), 1620-1623. DOI: 10.1016/j.tetlet.2012.01.070.
(5) Klein, J.; Seidel, A.; Frank, H.; Oesch, F.; Platt, K. L. Regiospecific oxidation of polycyclic aromatic dihydrodiols by rat liver dihydrodiol dehydrogenase. *Chem.-Biol. Interact.* **1991**, *79* (3), 287-303.
(6) Ozanne, A.; Pouységu, L.; Depernet, D.; François, B.; Quideau, S. A Stabilized Formulation of IBX (SIBX) for Safe Oxidation Reactions Including a New Oxidative Demethylation of Phenolic Methyl Aryl Ethers. *Org. Lett.* **2003**, *48*, 4605 -4609.
(7) Crich, D.; Zou, Y. Catalytic oxidation adjacent to carbonyl groups and at benzylic positions with a fluorous seleninic acid in the presence of iodoxybenzene. *J. Org. Chem.* **2005**, *70* (8), 3309-3311.
(8) Wu, A.; Duan, Y.; Xu, D.; Penning, T. M.; Harvey, R. G. Regiospecific oxidation of polycyclic aromatic phenols to quinones by hypervalent iodine reagents. *Tetrahedron* **2010**, *66* (12), 2111-2118. DOI: 10.1016/j.tet.2009.12.022.
